# Long non-coding RNAs are major contributors to transcriptome changes in sunflower meiocytes with different recombination rates

**DOI:** 10.1186/s12864-016-2776-1

**Published:** 2016-07-11

**Authors:** Nathalia M. V. Flórez-Zapata, M. Humberto Reyes-Valdés, Octavio Martínez

**Affiliations:** Laboratorio Nacional de Genómica para la Biodiversidad (LANGEBIO)/Unidad de Genómica Avanzada, Centro de Investigación y de Estudios Avanzados del Instituto Politécnico Nacional (Cinvestav), 36821 Irapuato, Guanajuato México; Department of Plant Breeding, Universidad Autónoma Agraria Antonio Narro, Buenavista, 25315 Saltillo, Coahuila México

**Keywords:** Meiosis, Prophase I, Homologous recombination, lncRNA, miRNA, RNA-seq, Transcriptomics

## Abstract

**Background:**

Meiosis is a form of specialized cell division that marks the transition from diploid meiocyte to haploid gamete, and provides an opportunity for genetic reassortment through recombination. Experimental data indicates that, relative to their wild ancestors, cultivated sunflower varieties show a higher recombination rate during meiosis. To better understand the molecular basis for this difference, we compared gene expression in male sunflower meiocytes in prophase I isolated from a domesticated line, a wild relative, and a F1 hybrid of the two.

**Results:**

Of the genes that showed differential expression between the wild and domesticated genotypes, 63.62 % could not be identified as protein-coding genes, and of these genes, 70.98 % passed stringent filters to be classified as long non-coding RNAs (lncRNAs). Compared to the sunflower somatic transcriptome, meiocytes express a higher proportion of lncRNAs, and the majority of genes with exclusive expression in meiocytes were lncRNAs. Around 40 % of the lncRNAs showed sequence similarity with small RNAs (sRNA), while 1.53 % were predicted to be sunflower natural antisense transcripts (NATs), and 9.18 % contained transposable elements (TE). We identified 6895 lncRNAs that are exclusively expressed in meiocytes, these lncRNAs appear to have higher conservation, a greater degree of differential expression, a higher proportion of sRNA similarity, and higher TE content relative to lncRNAs that are also expressed in the somatic transcriptome.

**Conclusions:**

lncRNAs play important roles in plant meiosis and may participate in chromatin modification processes, although other regulatory functions cannot be excluded. lncRNAs could also be related to the different recombination rates seen for domesticated and wild sunflowers.

**Electronic supplementary material:**

The online version of this article (doi:10.1186/s12864-016-2776-1) contains supplementary material, which is available to authorized users.

## Background

Meiosis is a complex cell division process that generates haploid gametes. During prophase I, the first and longest meiotic stage [1], chromosomes pair, synapse and recombine [2], which promotes increased genetic variation [3] and proper chromosome segregation in subsequent stages [4]. As expected, these meiotic events must be tightly coordinated. In yeast, the presence of transcriptional regulatory elements and temporary changes in gene expression highlight how transcriptional regulation may contribute to this coordination [5–7]. Several plants encode the transcriptional regulator gene *MMD1* [8, 9], while other genes such as the *LISCL* gene in lily and AMEIOTIC1 in maize are putative transcriptional regulators [10–12]. However, the transcriptional regulatory mechanisms involved in plant meiosis remain poorly understood [9, 13].

Advances in sequencing technologies and meiocyte collection techniques enabled the generation of transcriptomes for pure meiocytes in *Arabidopsis* [14, 15], maize [16], and sunflower [17]. These studies allowed the identification of new transcriptional regulatory elements [18, 19] and meiotic genes [20], and raise new questions about the transcriptional behavior of meiotic cells, particularly given the high levels of expression of transposable elements (TEs) in *Arabidopsis* meiocytes and mitochondrial genes in maize and *Arabidopsis* [14–16].

Additionally, the high transcriptional activity of meiocytes is remarkable. Around 20,000 genes in *Arabidopsis* and 30,000 in maize and sunflower are expressed [14–17]; these figures are comparable with the number of genes expressed in seedlings (which contain different tissues and cell types), suggesting that transcription during meiosis may be very promiscuous [13]. Nevertheless, many genes expressed in meiocytes correspond to unannotated features in the genome [19] or transcripts without a protein coding ortholog [17]. Some of these unannotated transcripts could be non-coding RNAs, especially since non-protein coding transcripts reportedly represent the majority of transcribed genes in eukaryote transcriptomes [21]. Non-coding RNAs (ncRNAs) are a diverse group of transcripts that includes housekeeping RNAs (e.g. ribosomal RNA, transfer RNA) and regulatory ncRNA [22]. Within the ncRNA regulatory group are long non-coding RNAs (lncRNA), which are >200 nt transcripts that do not encode a protein and can act as cis- or trans-regulators of gene transcription or as protein scaffolds in chromatin-modifying complexes [21, 23–25]. On the other hand, small RNAs (sRNA) are 20–27 nucleotide (nt) regulatory ncRNA that participate in post-transcriptional gene regulation and genome stability maintenance [26, 27]. Recently, sRNA and lncRNA were associated with the regulation of plant meiosis and fertility, although their specific function awaits clarification [28–31].

We previously showed that sunflower (*Helianthus annus* L.) is a good model for studying plant meiosis [17] because its inflorescence contains a large number of disk flowers that have different ages (growing older with the progression from the head center to the periphery) [32], which allows the isolation of nearly pure populations of male meiocytes in well-defined meiotic stages. In this study we sequenced the transcriptome of prophase I meiocytes from three different sunflower genotypes that were previously found to have significantly different recombination rates, inferred from chromosome pairing index in [33]. Interestingly, the largest proportion (~64 %) of differentially expressed genes (DEG) were not protein coding genes, but passed stringent filters to be classified as lncRNA. These lncRNAs are highly meiosis-specific and although some have sRNA-associated functions, others showed no connection with sRNA-mediated regulation, suggesting that lcRNAs may participate in other regulatory mechanisms. We propose that lncRNAs play a protagonist role in regulating meiotic gene expression or chromatin state changes during meiosis, which could also be related to the observed differences in the homologous recombination rate of the sunflower genotypes that we studied, as well as to other possible domestication-related meiotic traits.

## Results and discussion

### Differentially expressed genes between male sunflower meiocytes with different recombination rates

Chromosome pairing was significantly higher in a domesticated sunflower genotype (elite line HA89) relative to a wild sunflower genotype (Ac-8). Meanwhile, an F1 (F1) hybrid resulting from intercrossing these two genotypes had an intermediate rate of chiasmate chromosome arms [33]. The effect of domestication on the recombination rate was previously documented by Ross-Ibarra [34], who observed that domesticated plants have higher recombination rates than their wild relatives, and proposed that domestication selects for this increased recombination. As a first approach to understand which genes or regulatory processes could be related to variations in chiasma frequency, we conducted a transcriptome analysis of sunflower prophase I meiocytes from Ac-8 (wild type, *H. annuus ssp. texanus*), HA89 (domesticated elite line, *H. annuus* var. *macrocarpus*), and an F1 hybrid of the two, which correspond to the genetic materials used in the previous report of comparative analysis of chiasmate chromosome arms [33].

We obtained ~8.6 × 10^8^ pair-end reads for the sunflower meiocytes (see Methods and Table AF1-1 in Additional file 1). The F1 genotype reads were quality-trimmed and used for a *de novo* transcriptome assembly, which was used as a reference transcriptome for subsequent analyses (see section “Sequencing and assembly results” in Additional file 1). Around 78 % of the reads (Table AF1-1 in Additional file 1) mapped to a unique position within one of the 73,658 distinct transcripts (“genes”) in this transcriptome. More than half the transcripts (39,354, 53.42 %) were annotated *via* BLAST with any of the peptide databases queried (see Methods), while the remaining 34,304 transcripts (46.58 %) could not be identified using this approach. Identified transcripts that shared the same BLAST identifier were considered to be either products of the same sunflower locus or derived from closely related paralogs. To quantify the expression of these transcripts, reads aligned to these loci were added and the transcripts were “collapsed” to treat the related transcripts as a single gene. The final dataset included 59,085 genes.

To estimate how many genes could be missing from our sample, we applied the method described by García-Ortega and Martínez [35]. The estimate for the number of missing genes in our dataset was equal to zero, and the 95 % confidence interval for the number of missing genes was zero to three, indicating that, within the total sample, our RNA-seq experiment detected practically all expressed transcripts and thus no extra sample was needed to detect missing genes. This result implies that genes detected only in meiocytes and not in somatic tissues are likely to have exclusive meiocyte expression.

**Fig. 1:**
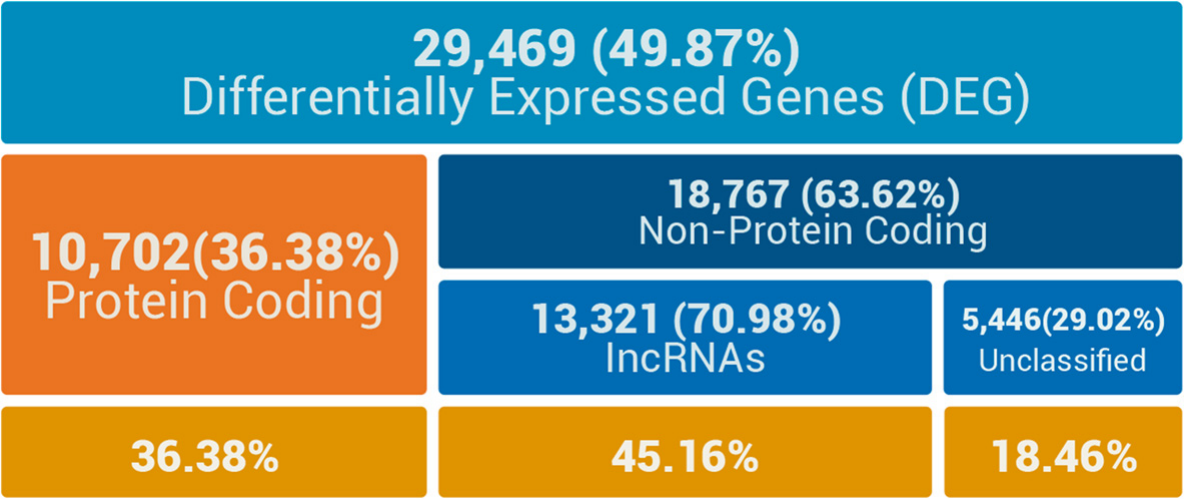
Diagram showing the numbers and proportions of differentially expressed genes (DEG) between the domesticated and wild genotypes grouped by coding class. Global percentage of DEGs (first row) was calculated based on the total number of genes (59,058). Percentages of genes with and without protein coding potential were calculated with reference to number of DEGs (29,469). Percentages of lncRNA and unclassified genes were calculated with reference to non-protein coding genes (18,767). Last row in the diagram presents percentages in each category with reference to the total DEG number (29,469)

We found 29,469 (49.87 %) differentially expressed genes (DEGs) between the domesticated and wild genotype using a False Discovery Rate (FDR) of 1 %. The majority (63.62 %) of these genes could not be identified via BLAST (Fig. 1), which is consistent with our previous study wherein transcripts that could not be identified were more abundant in meiocytes than in the somatic transcriptome, and also exhibited more tissue-specific expression [17]. Thus, we focused on these unidentified transcripts to investigate how many could be classified as lncRNAs that may play a regulatory role in homologous recombination during meiosis.

### lncRNA identification in sunflower meiocytes

**Fig. 2:**
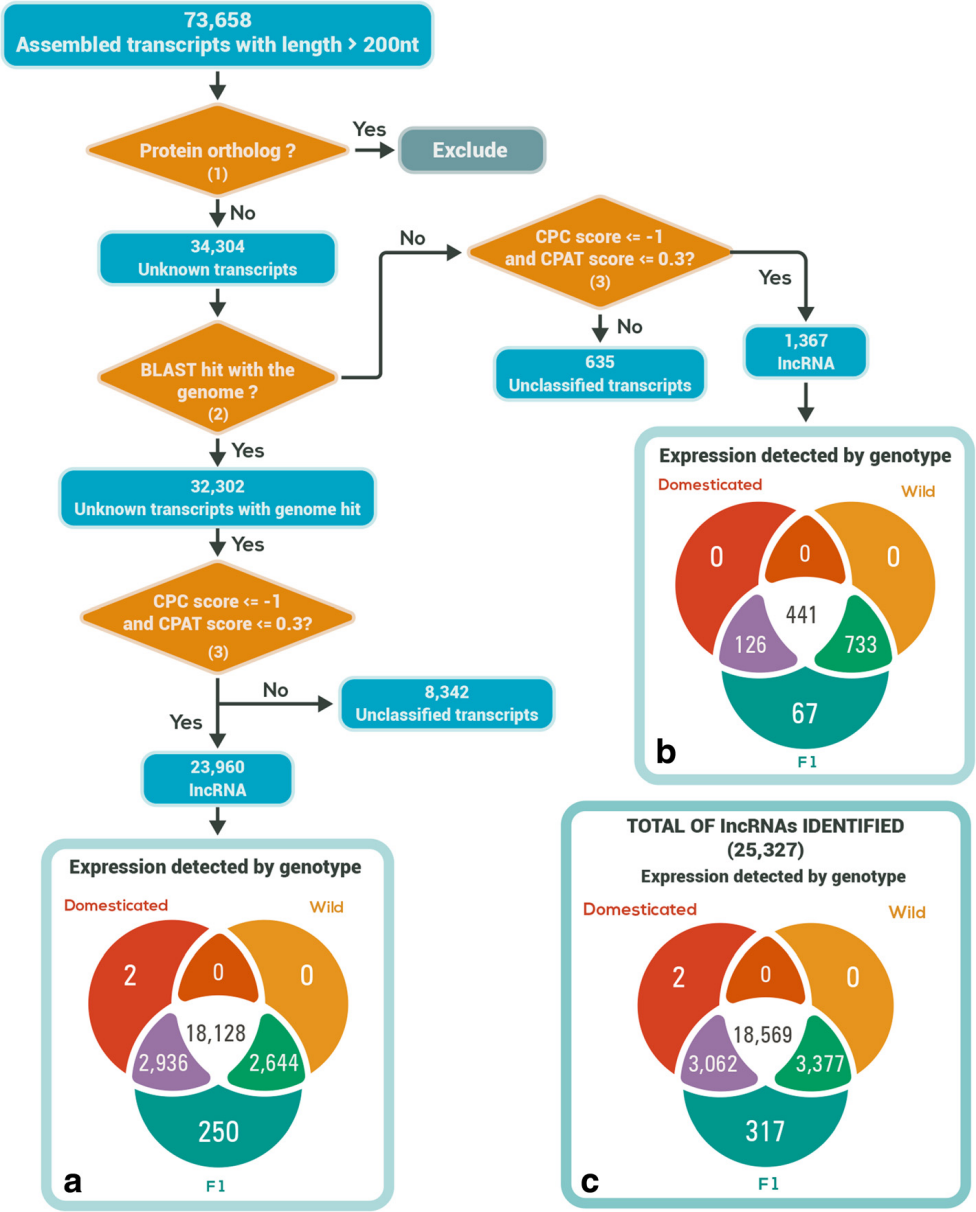
Bioinformatic pipeline to determine the lncRNA nature of transcripts and Venn diagrams with number of lncRNA expressed by genotype. Protein coding was determined by comparing transcripts with peptide databases (TAIR 10, NCBI RefSeq and sunflower peptides); all transcript with one or more hits to peptides with a bitscore ≥ 90 and E value < 1e-6 were discarded as potential lncRNAs in (1). To determine if the transcripts had a blast hit with the draft of the sunflower genome, a threshold of bitscore ≥ 90 and E-value < 1e-6 was employed in (2). Only transcripts with a CPC score ≤ −1 and CPAT score ≤ 0.3 were considered as lncRNA by filters in (3). Venn diagrams: **a**) Expression of lncRNAs with significant similarity with the sunflower genome. **b** Expression of lncRNAs with no significant similarity with the sunflower genome. **c** Total number of lncRNAs detected

To test if the unidentified transcripts expressed in sunflower meiocytes were lncRNAs, we used the workflow described in Fig. 2. First, we excluded all 39,354 transcripts with protein coding potential using BLAST with protein databases (see Methods). For the remaining 34,304 unknown transcripts we performed a BLASTN search against the draft genome sequence compiled by the Sunflower Genome Project (in progress for the inbred line HA412). Among these transcripts, 90.88 % had a BLAST hit that was more than 90 % identical to the genome draft. This result suggests that most of these sequences were indeed sunflower transcripts and not assembly artifacts. On the other hand, discarding the transcripts that lacked a genome hit would be unwise, as they may be products of RNA processing or genotype-specific sequences. Thus, we tested both unknown transcripts (with and without genome hit), for their protein coding potential with two different algorithms: CPC (Coding Protein Calculator) [36] and CPAT (Coding-Potential Assessment Tool) [37], and only those transcripts that passed the thresholds of both algorithms were classified as lncRNAs (See Methods). Given that the methods used by CPC and CPAT are complementary (CPC uses a support vector machine classifier, while CPAT employs a logistic regression model), the classification of sequences as lncRNA only when both algorithms concurred can be considered highly trustworthy (see “Additional discussion of lncRNA Identification” in Additional file 1).

Of the unknown transcripts with and without a genome hit, 74.17 % (23,960) and 68.28 % (1367), respectively, were classified as lncRNAs (Fig. 2). Other transcripts that did not pass one or both of the coding potential filters (CPC and CPAT) were designated as unclassified. We examined the expression of the lncRNA-classified transcripts in the three sunflower genotypes, and found that 75.65 % (18,128) of the lncRNAs with a genome hit showed evidence of expression in all three sunflower genotypes tested, so these can be considered as highly reliable sunflower lncRNAs (Fig. 2a). On the other hand, 32.26 % (441) of the lncRNAs without a genome hit showed expression in all three sunflower genotypes (Fig. 2b).

In contrast, the proportion of lncRNA with a genomic hit detected in the F1 and wild genotypes was only 11.03 % (2644 of 23,960), while those without a genomic hit represented approximately 53.62 % (733 of 1367) of the total. This enrichment in the ‘wild origin’ lncRNA without a genome hit may be to due to our use of a domesticated genotype (inbred line HA412) as a reference.

A Venn diagram of all lncRNAs showed that all but two (>99.99 %) were detected in the F1 hybrid, and the remaining two lncRNAs appeared only in the domesticated genotype (Fig. 2c). The majority (73.31 %; 18,569) of the lncRNAs were detected in the F1 and both parental genotypes (inner intersection), while similar proportions were detected in the F1 and one of the parental genotypes (12.08 % (3062) and 13.33 % (3377) for F1-domesticated and F1-wild, respectively). Interestingly, 317 (1.25 %) of the lncRNAs were exclusively detected in the F1. This expression pattern suggests that processing of the original transcript to a mature lncRNA could be affected by the interaction between the wild and cultivated genomes. After transcription, most lncRNAs are processed similarly to protein-coding RNAs, including 5'-end capping, 3'-end polyadenylation, and splicing modifications [38]. Although alternative splicing appears to be less common in plants than in animals, in grape plants expression of alternative spliced forms is reportedly genotype-dependent [39].

Among the 25,327 lncRNAs identified, 13,321 showed differential expression between the domesticated and wild genotypes. qRT-PCR analysis of a selected set of differentially expressed lnc-RNAs was performed. For the five lncRNAs where the qRT-PCR were completed, the tendencies in fold change between the domesticated and wild genotypes were validated; see section “qRT-PCR analysis of selected lncRNAs” in Additional file 1 for details. The proportion of DEG lncRNAs and unclassified transcripts was significantly higher than the proportion of protein-coding DEGs (45.16 % and 36.38 %, respectively, *P* < 0.01) (Fig. 1). This result indicates that the major changes observed at the transcriptomic level between meiocytes of domesticated and wild origin are due to changes in lncRNA gene expression, which could be related to the differential recombination rates between these genotypes. A recent study by Ding et al. [40] highlighted the importance of lncRNAs in fission yeast meiosis by demonstrating that the *sme2* gene that encodes a meiosis-specific lncRNA is important for homologous recognition and homologous chromosome pairing. Although no meiosis-specific lncRNA are yet known in plants [13], many lncRNAs are associated with sexual reproduction and fertility. For example, the maize lncRNA *Zm401* is thought to be essential for tapetum and microspore development [41], while rice LDMAR lncRNA regulates photoperiod-sensitive male sterility and normal pollen development [42]. Indeed, a large number of lncRNAs important for sexual reproduction in rice have been identified through genome wide screening [29].

Transcriptome analysis of mammalian testes showed that the gene expression levels in this organ are higher relative to other organs (e.g., brain, heart, liver, kidney), and this difference is more pronounced for predicted lncRNAs, which have higher expression in testis than in other organs [43]. Concordantly, we previously demonstrated that in humans the testis has the highest transcriptome diversity [44]. Moreover, the repertoire and expression pattern of lncRNAs in tetrapods showed that lncRNAs are preferentially expressed in the testes, and this expression is actively regulated, which suggests that this expression is not due only to non-specific transcription in open chromatin regions [45]. The co-expression networks of lncRNAs with protein-coding genes showed that the clusters with the highest lncRNA proportions were enriched in spermatogenesis functions, which is in agreement with the high proportions of lncRNAs in the testes, as well as the substantial contribution that pachytene spermatocytes make to the transcriptome of whole testes [43, 45]. On the other hand, a genome-wide characterization of maize lncRNAs showed that male reproductive tissues such as immature tassels, anthers, and pollen, had higher lncRNA levels than did other tissues [46]. Thus, this lncRNA enrichment in animal and plant reproductive tissues during meiosis and gametogenesis may arise from a conserved and well-structured regulation of gene expression that involves lncRNAs.

The molecular mechanisms by which lncRNAs could participate in plant meiosis are not well understood. According to their function in other biological processes, they may play roles in controlling gene expression, influencing epigenetic factors, maintaining characteristics of heterochromatin, or controlling transposable elements [13]. In rice, more than 700 lncRNAs appear to be key factors for inducing the biogenesis of 21 nt phased siRNAs (phasiRNAs) that are associated with the germline-specific MEL1 argonaute protein [47], implying that in meiosis lncRNAs could also act as precursors or mimics of sRNA targets [48]. In addition, lncRNAs could play a direct structural role similar to the human skin fibroblast cell line lncRNA *DDSR1*, which interacts with BRCA2 to modulate DNA repair by homologous recombination [49], a repair pathway that is also important for proper meiosis in *Arabidopsis* [50].

Differences in lncRNA expression among genotypes and consequent phenotypic effects of these differences have been observed in wheat, wherein the expression of two lncRNAs (*TalncRNA73* and *TalncRNA108*) in three genotypes is closely related to stripe rust susceptibility [51]. Meanwhile, two porcine lncRNAs (*linc-sscg2561* and *Dnmt3a*) showed differential expression levels between domesticated pigs and wild boars, suggesting a possible role for lncRNAs in pig domestication [52]. Maize domestication also reportedly reshaped the transcriptome, since DEGs are enriched in targets of selection during maize domestication and improvement [53]. Therefore, the differentially expressed lncRNAs we identified here could have important regulatory functions in meiosis, and, given the effect that the selection process has on recombination, may have been subjected to selection through sunflower domestication [34].

### Comparing the expression of lncRNAs in somatic and meiocyte transcriptomes

**Fig. 3:**
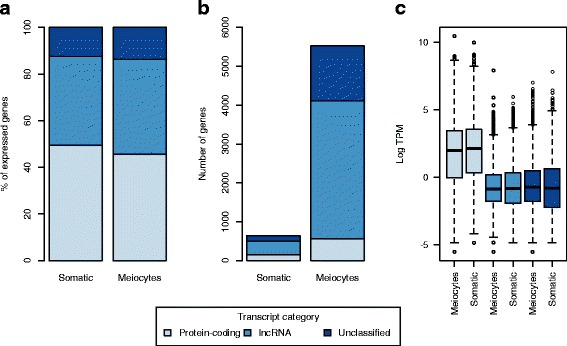
Expression overview of genes grouped by coding class (Protein-coding, lncRNA or Unclassified) in somatic and meiocyte transcriptomes. **a** Bar chart showing the coding class proportions by transcriptome. **b** Bar chart comparing the number of genes exclusively expressed in somatic or meiocyte transcriptomes by coding class. **c** Box plots for the distributions of the decimal log expression of genes in meiocytes and somatic transcriptomes by coding class

To gain insight into the function of sunflower lncRNAs, we examined their expression behavior in the meiocyte and somatic transcriptomes of the domesticated sunflower genotype HA89 [17]. In HA89 meiocytes, the proportion of lncRNAs was significantly higher (*P* < 0.01) than that of the somatic transcriptome (Fig. 3a). Likewise, as mentioned above, high expression levels of lncRNAs in reproductive structures have been described in plants [46] and animals [43, 45]. Given the high number of lncRNAs expressed in sunflower meiocytes, many are likely involved in meiosis, and therefore the expression (and possible role) of lncRNAs in meiosis could be highly conserved.

Furthermore, we found that the majority of transcripts that showed expression in only one of the two transcriptomes (exclusively expressed genes), were lncRNAs in both somatic and meiocyte transcriptomes (Fig. 3b), although the number of lncRNAs was higher for the meiocyte transcriptome. On the other hand, the expression level (in transcripts per million; TPM) of these lncRNAs was lower than that observed for protein-coding genes (Fig. 3c), suggesting that sunflower lncRNAs are also very tissue-specific and have lower expression levels than protein-coding genes. These two common characteristics of lncRNAs are consistent with findings for other organisms [21, 29, 46].

### Small RNA (sRNA) populations in sunflower meiocyte transcriptomes and their relationship with meiotic lncRNAs

According to Li et al. [46], more than the 90 % of putative maize lncRNAs have sequence similarity with small RNAs (sRNA), which they classified as pre-lncRNAs. Additionally, in other model species such as rice, *Arabidopsis,* and *Populus trichocarpa*, lncRNAs could be precursors [47, 54] or mimic targets of sRNAs [48, 55]. Thus, to characterize the connection between non-coding sRNAs and lncRNAs, we sequenced the sRNA transcriptome of prophase I meiocytes from wild and domesticated sunflower genotypes.

**Fig. 4:**
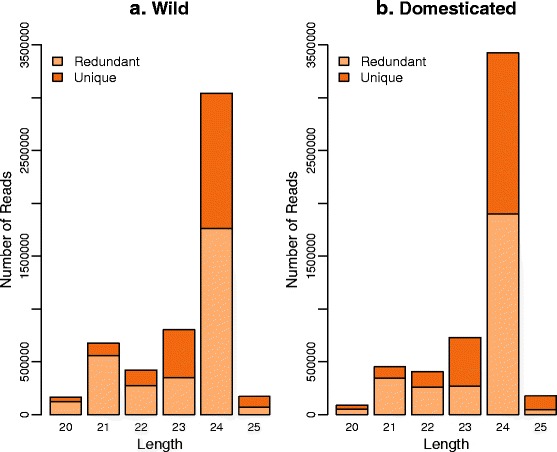
Bar charts for the number of reads per length (bp) in small RNA populations of sunflower meiocytes in two genotypes. Reads were classified as ‘Unique’ for sequences found only once, or ‘Redundant’ for sequences found more than one time within genotypes. **a** Results for the wild genotype Ac-8. **b** Results for the domesticated genotype HA89

We obtained around 5 million (Table AF1-2 in Additional file 1) clean reads of 20 to 25 nt, with most corresponding to reads of 24 nt (Fig. 4). These 24 nt sRNAs are typically endogenous siRNAs [27] and are the major component of sRNA populations in plants that participate in RNA-mediated chromatin-based gene silencing [56]. During maize meiosis, 24 nt phasiRNAs accumulate [30] in a way that is similar to the accumulation observed for mouse spermatogenesis [31]. Although the function of these 24 nt sRNAs is not completely understood, they may participate in genome surveillance (e.g., TE silencing pathways), or act as mobile signals and/or chromatin modifiers [30, 31].

We previously found that gene silencing pathway genes are highly expressed in meiocytes, especially the ortholog of the *Arabidopsis* AGO4 protein, which participates in 24 nt siRNA binding. We found that meiocyte expression of the sunflower AGO4 ortholog was increased by 2.9-fold relative to the somatic transcriptome [17]. Given that *Arabidopsis* AGO4 is known to preferentially bind 24 nt siRNAs with a 5' adenosine [57], we examined 5' terminal nucleotide bias in sunflower 24 nt sRNAs. A majority of sunflower 24 nt sRNAs indeed had a 5' terminal adenosine (Figure AF1-2 in Additional file 1), suggesting that these 24 nt sRNAs could be loading in the sunflower AGO4 orthologous complex, and thus could participate in maintaining silent states at repeated loci, transposons, and heterochromatin, in a similar manner to that of *Arabidopsis* [58]. The description of these abundant 24 nt sRNAs in sunflower meiocytes helps to support the idea of a convergent evolution of sRNA systems that regulate male reproductive development [30, 31].

Consistent with observations in other plant species [59–61], the 24 nt sRNA population in sunflower is less redundant than the 21 nt population (Fig. 4). sRNA with 24 nt have a higher number of unique or low abundance reads, while the 21 nt population includes multiple copies of the same sRNA. Moreover, the 21 nt sRNA population in plants is usually composed of miRNAs, which help regulate gene expression through post-transcription gene silencing mechanisms [62].

The expression of 32 different miRNA families was detected in both wild and domesticated genotype meiocytes (Figure AF1-3 in Additional file 1), but 92.69 % and 85.87 % of the reads for the domesticated and wild genotypes, respectively, were one of only three different 21 nt miRNA (miR166, miR396, and miR319) that show high conservation among terrestrial plant species [62]. Although the function of these three miRNAs in meiosis is unknown, in tomato miR396 and miR166 are differentially expressed between wild type and male-sterile mutant *7B-1* anthers, suggesting that they may have a role in anther development and male fertility [63]. Meanwhile, miR319 is expressed in the *Arabidopsis* male germline [64]. Here the relative abundance of these miRNA families was almost the same in the wild and domestic genotypes (Figure AF1-3 in Additional file 1), with the exception of miR398, which was 7-fold more abundant in the wild meiocytes. In cotton, miR398 was also differentially expressed during meiosis and tetrad stages of anthers from wild type and genetic male sterility (GMS) mutant plants [65]. The above finding indicates that the role of mi398 in meiosis merits additional study.

**Fig. 5:**
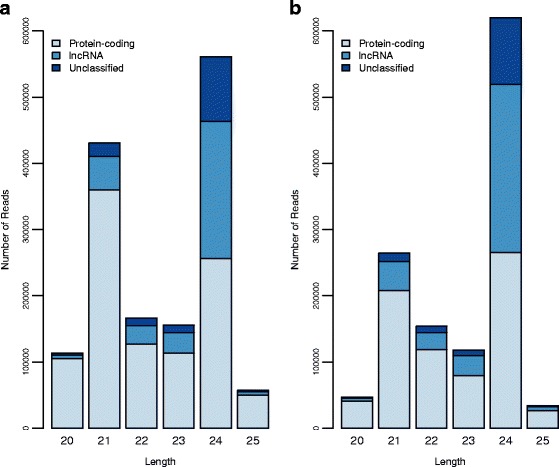
Bar charts for the number of reads per length (bp) in small RNA populations of sunflower meiocytes mapping to protein-coding, lncRNA or unclassified transcripts in two genotypes. **a** Results for the wild genotype Ac-8. **b** Results for the domesticated genotype HA89

To establish the relationship between sRNA populations and lncRNAs, we mapped sRNAs against a mixed reference (contigs of the genome draft and the transcriptome assembled for this study), which allowed us to avoid selection bias, that is, to assign a transcript as a precursor or target of a sRNA, when the best hit for that sRNA is in a non-transcribed intergenic region (See Methods). Most 21 nt sRNAs mapped to protein coding transcripts, while the proportion of 24 nt that mapped to protein-coding transcripts was similar to that which mapped to lncRNAs (Fig. 5). sRNA reads of the wild genotype mapped to 9370 lncRNAs, while the domesticated genotype sRNA reads mapped to 8852 lncRNAs (42.69 % and 40.91 %, relative to the total lncRNAs expressed in each genotype). sRNAs reads for both genotypes mapped to the same 8852 lncRNAs. Even though the proportion of lncRNAs having sequence similarity with miRNAs is notable, it is not as high as that for maize [46].

According to Axtell [27], the patterns of reference-aligned sRNAs provide information about sRNA biogenesis. We found that 20 and 21 nt sRNAs showed major differences (~15 % more reads mapped to the ‘+’ strand) in the proportion of reads that mapped to the lncRNA ‘+’ or ‘-‘ strand (Figure AF1-4 in Additional file 1). This result is expected, considering that these sRNA sizes are associated with miRNAs, which are generated through fragmentation of a single-stranded precursor, while 24 nt (mostly siRNAs) arise from double-stranded RNA precursor cleavage. Thus, siRNAs are indeed the major sRNA population in sunflower meiocytes, and many lncRNAs are siRNA precursors.

On the other hand, according to BLAST searches against sunflower natural antisense transcripts (NATs) in the PlantNATs public database (See Methods), we determined that 388 lncRNAs are trans-natural antisense transcripts (trans-NATs). Of these trans-NATs, 198 and 215 for domesticated and wild type genotypes, respectively, could be cataloged as sRNA precursors, since sRNAs reads mapped to these NATs. Furthermore, 68.37 % and 68.68 % of NATs for wild and domesticated genotypes, respectively, are related to 24 nt sRNAs. Other sRNA lengths were also related to NATs, albeit in lower proportions (Figure AF1-5 in Additional file 1). Thus, some NAT-siRNAs are also active in sunflower meiocytes and could be part of regulatory mechanisms for gene expression that involve transcript cleavage, as was previously described for these types of sRNA [66, 67].

Together our results indicate that there is a complex regulatory network of sRNAs-lncRNAs working at the transcriptomic level in sunflower meiocytes. Considering that various sunflower genotypes show differences in lncRNA expression, lncRNAs are likely relevant to the regulation of recombination rates. However, some lncRNAs appear to be unrelated in sRNA regulatory pathways, so they may be involved in regulating transcription through other mechanisms.

### Repetitive elements in sunflower lncRNAs

Transposable elements (TEs) are thought to be important contributors to the origin, evolution, and function of lncRNAs [68]. Through analysis of different regions of long intergenic non-coding RNAs (lincRNA) in mice and humans, Kannan et al. [69] found that the TE content of lincRNA genes is higher than in protein-coding genes, and that most TEs are present in the exons and promoter regions of lincRNAs. They also observed a correlation between TE insertion and the evolutionary rate of lincRNAs (e.g., there was more TE fixation in fast-evolving lincRNA genes). On the other hand, in tomato the insertion of a long terminal repeat (LTR) retrotransposon was important in the origin of the fruit-specific lncRNA *lncRNA-314*, which presumably was generated during tomato domestication given its potential involvement in fruit ripening [70].

**Fig. 6:**
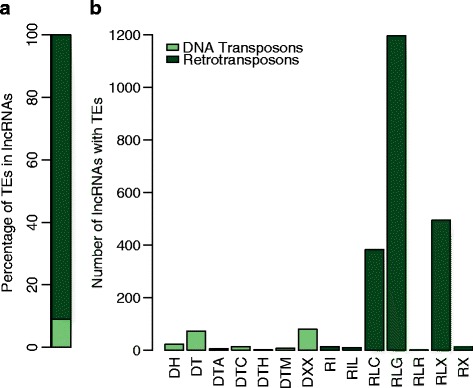
Transposable elements (TEs) in sunflower lncRNAs. **a** Relative percentages of DNA Transposons and Retrotransposons found within 2326 (9.18 %) sunflower lncRNA. **b** Bar chart for the number of lncRNA containing TEs per TEs family. DNA Transposons: DH = Helitron, DT = Unknown DNA transposon, DTA = hAT, DTC = CACTA, DTH = Harbinger, DTM = Mutator, DXX = MITE. Retrotransposons: RI = unknown non-LTR retrotransposons, RIL = LINEs, RLC = Copia retrotransposons, RLG = Gypsy retrotransposons, RLR = retroviruses, RLX = Unknown LTR retrotransposons, RX = Unknown retrotransposons

We found that 2326 (9.18 %) of all lncRNAs identified here contained TEs (Fig. 6). Of these, 91.01 % also carried retrotransposons, while the remaining 8.99 % had DNA transposons (Fig. 6a). The most common TE belonged to the long terminal repeat (LTR) retrotransposons from the *Gypsy* and *Copia* families (Fig. 6b). These results are consistent with previous studies showing that these two retrotransposon families are also the more abundant in the sunflower genome [71, 72]. Other repetitive elements, such as tandem repeats and unknown repeats, were also identified (Figure AF1-6 in Additional file 1). The percentage of lncRNAs that contained transposable or repetitive elements was 14.31 %, which was lower than that for maize [46]. Although the different lncRNA TE content in maize and sunflower could be due to methodological strategy, they may also be related to differences in genomic TE contents of the tissues from which the lncRNAs were sampled (i.e., meiotic expressed lncRNAs in sunflower vs. whole-plant expressed lncRNAs in maize), or intrinsic characteristics of lncRNAs. Even in maize, the majority of so-called HC-lnRNAs (those that are not sRNA precursors) do not contain repetitive sequences [46], which suggests that lncRNA diversity occurs at both functional and evolutionary levels, even within the same species. As such, inter-species differences in lncRNA characteristics would certainly be expected.

The presence of TEs in sunflower lncRNAs is an interesting result, since these elements could contribute to an understanding of their function and biology. For instance, TE-related variability was reduced among domesticated plants compared to wild *H. annuus* accessions [72]. Moreover, the involvement of TE in domestication and trait improvement has also been reported for several plants, including maize and wheat [73], indicating that the TE content between wild and domesticated plants is dynamic. Thus, the domestication process can create a permissive environment for the generation of new or altered lncRNAs that in turn affect regulatory networks by fostering phenotypic changes (e.g., differences in the recombination rate) in a relatively short timeframe.

One surprising finding of transcriptome studies in *Arabidopsis* meiocytes [14, 15] was the high expression of TEs, which could generate mutations or have harmful effects on the genome or germline that could be inherited by future generations [13]. Thus, maintenance of the structural integrity of a plant’s genetic material is critical. Although transposon expression may be an unintended result of chromatin structure reorganization during meiosis [13], their expression could also have a functional role, especially since TEs can participate in chromatin remodeling by recruiting silencing machinery [74]. Thus, although some TE expression in meiocytes is likely genuine, which would be justified by the high expression of proteins such as AGO9 that participate in TE silencing mechanisms during formation of female gametes [75] and the siRNA silencing pathways described above [30, 31], some meiocyte TEs could actually be functional domains of lncRNAs [68]. This possibility implies that TEs perform their role in chromatin remodeling during meiosis, and their integration in lncRNAs would not compromise genomic integrity. Even though future analyses are needed to test this hypothesis, the presence of TEs in lncRNAs that are highly expressed in meiocytes opens a new perspective that could yield clues about their highly counterintuitive expression in the germ line.

### Identification of putative targets for lncRNAs

The identification of molecules that interact with the lncRNAs, could be also important to elucidate their function. One of the possible functions of lncRNAs is regulation of protein-coding transcripts through RNA/RNA interactions [24]. With the aim of predicting putative protein-coding targets for the set of lncRNAs found in this work, we tested the ‘LncTar’ algorithm [76], on a subset of lncRNAs and sunflower meiotic genes. In this experiment we found some promising results, as the fact that three lncRNAs have a predicted significant interaction with sunflower meiotic gene MMD1, involved in spindle cytokinesis, and also these three lncRNA present a highly correlated change of relative expression with MMD1 in the three genotypes (*r* > 0.98; see Tables AF1-6 and AF1-7 and Figure AF1-1 in section “Putative Targets for lncRNAs” in Additional file 1). Given that *in silico* prediction of RNA/RNA interaction, as detected by LncTar, is only one of the factors to take into account to predict lncRNA/target interactions, the above example and many more detected (see “Putative targets for lncRNAs” in Additional file 1), need further experimental confirmation to be taken into account in the understanding of individual lncRNAs functions.

### Final remarks on sunflower meiotic lncRNAs

The idea that lncRNAs act as key gene regulators is supported by several features such as: i) immediate functionality upon transcription (i.e., no need for translation into protein); ii) versatile structural or sequence-specific interactions with proteins or nucleic acids; and iii) evolutionary flexibility that promotes tolerance of insertions or deletions given that their function is independent of a strict sequence frame like that of protein-coding genes [68]. These features are consistent with increasing evidence suggesting that lncRNAs are involved in many different biological processes, and thus expand our knowledge of the regulation of certain processes and/or alter some paradigms [21, 25]. A meeting point of studies describing genome-wide identification of lncRNAs is that lncRNAs are highly expressed in sexual reproduction structures in both animals [45] and plants [46]. Although the relationship of lncRNAs to plant fertility is also established [29, 41, 42], as is the induction of 21 nt phasiRNAs in rice meiosis [47], no plant meiosis-specific lncRNA has yet been described [13]. So, to the best of our knowledge, this is the first report of meiosis-specific lncRNAs in plants, which open questions about their function and importance in regulating fundamental sexual reproduction processes.

To explore the possible role of these meiosis-specific lncRNAs in sunflower meiocytes, we compared them with lncRNAs expressed in the somatic transcriptome (Table 1). The meiosis-exclusive lncRNAs had significantly more hits in the sunflower genome (87.18 % vs. 64.77 %, *P* < 0.01), greater differential expression (58.22 % vs. 50.49 %), a larger degree of sRNA similarity (36.77 % vs. 23.33 %), and a higher TE content (8.22 % vs. 6.46 %) than lncRNAs expressed in both meiocyte and somatic transcriptomes. These meiocyte-specific differences give valuable clues about lncRNA function in meiosis. First, the larger number of genome hits suggests greater conservation, which is in agreement with previous observations in tetrapods, where lncRNAs related to spermatogenesis displayed a higher level of conservation [45]. Second, some meiosis-specific lncRNAs could play a larger role in the recombination rate relative to non-specific lncRNAs. Finally, their higher similarity with sRNAs and higher TE content suggest that many lncRNAs may promote chromatin state modifications, especially given the differences in chromatin reorganization during meiosis [43, 77, 78] and that sRNAs and TEs are thought to have regulatory roles in chromatin modification [74, 79].

**Table 1:** Summary of sunflower lncRNA features. The total number of lncRNAs having each feature is presented. Percentages of differentially and non-differentially expressed lncRNAs were calculated according to the total of lncRNAs with a given feature (values in the total column). Percentages in the total for each feature (total column) were calculated with respect to the total number of lncRNAs independently of features (value in the total row and total column)

	Not differentially expressed	Differentially expressed	Total
Somatic and meiocytes expressed			
Genome hit	8925 (49.72 %)	9024 (50.28 %)	17,949 (70.87 %)
Expression in three genotypes	8250 (52.65 %)	7421 (47.35 %)	15,671 (61.87 %)
Expression in three genotypes and genome hit	8083 (52.80 %)	7227 (47.40 %)	15,310 (60.45 %)
sRNAs similarity	3035 (44.40 %)	3800 (55.60 %)	6835 (26.99 %)
Contains transposons	831 (47.25 %)	928 (52.75 %)	1759 (6.94 %)
Total of lncRNAs identified	12,006 (47.40 %)	13,321 (52.60 %)	25,327
Meiocytes 'exclusive'			
Genome hit	2605 (43.34 %)	3406 (56.66 %)	6011 (87.18 %)
Expression in three genotypes	1564 (53.97 %)	1334 (46.03 %)	2898 (42.03 %)
Expression in three genotypes and genome hit	1520 (53.94 %)	1298 (46.06 %)	2818 (40.87 %)
sRNAs similarity	1081 (42.64 %)	1454 (57.36 %)	2535 (36.77 %)
Contains transposons	279 (49.21 %)	288 (50.79 %)	567 (8.22 %)
Total of meiocytes 'exclusive' expressed	2881 (41.78 %)	4014 (58.22 %)	6895
Somatic and meiocytes expressed (meiocytes 'exclusive' not included)			
Genome hit	6320 (52.94 %)	5618 (47.06 %)	11,938 (64.77 %)
Expression in three genotypes	6686 (52.34 %)	6087 (47.66 %)	12,773 (69.33 %)
Expression in three genotypes and genome hit	6563 (52.54 %)	5929 (47.46 %)	12,492 (67.77 %)
sRNAs similarity	1954 (45.44 %)	2346 (54.56 %)	4300 (23.33 %)
Contains transposons	552 (46.31 %)	640 (53.69 %)	1192 (6.46 %)
Total of lncRNAs expressed in somatic and meiocyes, excluding meiocytes 'exclusive' expressed	9125 (49.51 %)	9307 (50.49 %)	18,432

We also observed differences in lncRNA expression between domesticated and wild genotypes. In lncRNAs with exclusive expression, the proportion of non-differentially expressed lncRNAs with genome hits was lower than that for differentially expressed lncRNAs (43.34 % vs. 56.66 %), while for non-exclusive lncRNAs the opposite tendency was observed (higher proportion in non-differentially expressed lncRNAs, 52.94 % vs. 47.06 %). These differences may provide evidence that meiocyte-specific lncRNAs could be more closely related to differences in the recombination rate than non-meiocyte specific lncRNAs, and that the major proportion of these lncRNAs are more conserved or do not have splicing modifications (which explains the higher number of genome hits). We also found that lncRNAs with differential expression have higher sRNA similarity in both meiocyte-specific and non-meiocyte-specific lncRNAs, although this difference is more notable for the specific lncRNAs (57.36 % vs. 42.64 % and 54.56 % vs. 45.44 % for meiocyte-specific and non-meiocyte specific lncRNAs, respectively). Since lncRNAs with more variable expression were mostly related to sRNAs, the linked chromatin modification function of lncRNAs-sRNAs may provide the most significant contribution to the differences in recombination rates. Lastly, differences in the proportion of differentially and non-differentially expressed lncRNAs that contain TEs were observed. In meiocyte-specific lncRNAs the proportion of non-differentially expressed lncRNAs with TEs was slightly lower than that for differentially expressed lncRNAs (49.21 % vs. 50.79 %). However, for non-meiocyte-specific lncRNAs we observed that this difference is wider (46.31 % vs. 53.69 %, non-differentially and differentially expressed lncRNA, respectively).. Further studies will be needed to clarify the role of these TE-containing lncRNAs in meiosis, and also to establish if their function is related to the higher TE frequency in meiocytes [14, 15].

## Conclusions

In recent years growing evidence suggested that lncRNAs have a wide range of important regulatory functions [21–23, 38]. In particular, genome-wide identification and characterization of lncRNAs in different plant and animal species spotlighted their high expression in reproductive structures such as testicles and anthers [29, 43, 45, 46], and that these lncRNAs have a greater degree of conservation that is suggestive of their key role in sexual reproduction [45]. Although the involvement of lncRNAs in sexual reproduction and fertility in plants [29, 41, 42] is well documented, whether lcRNAs also function in plant meiosis was unclear [13], and no plant meiosis–specific lncRNAs were known. Here, we obtained transcriptomes of meiocytes in prophase I from three different sunflower genotypes that showed different recombination rates. Through sequencing we could obtain a complete transcriptome with no missing genes [35], with which we generated results indicating that a major proportion of DEGs were lncRNAs, some of which were expressed exclusively in meiocytes.

Our data suggest that, relative to non-meiosis specific lncRNAs, meiosis-specific lncRNAs are more conserved within related genotypes (a major proportion had a genome hit), have greater expression variability in meiocytes from wild and domesticated genotypes, and have a closer relationship with elements such as siRNAs and TEs that are related to chromatin remodeling. These results highlight the fundamental role of lncRNAs in meiosis, and suggest a connection between two features of meiocytes and/or anthers: high TE activity and higher frequency of siRNAs. We also found evidence to support a role for lncRNAs in meiotic functions [13], such as maintenance of heterochromatin and influencing epigenetic factors.

Although whether lncRNAs directly or indirectly affect meiotic homologous recombination, or whether other factors could explain the differences in the recombination rate is unclear, the meiosis-specific differentially expressed lncRNAs identified here may be involved in processes that led to these phenotypic differences. For instance, some lncRNAs are associated with the advent of domestication features [52] or were modified during domestication [70], suggesting that the strong artificial selection that occurs during domestication could influence lncRNAs. Likewise, lncRNAs are both widely conserved and rapidly evolving elements [45, 46, 80], and therefore may represent a rich source for evolutionary innovations [79] that allow greater flexibility in selection processes without compromising sexual reproduction, or for regulatory changes in meiosis that do not affect essential genetic elements.

Future efforts will focus on determining whether sunflower-specific or preferentially expressed lncRNAs are conserved in other plant species, and in characterizing their activity at a functional level. These studies will require the generation and re-analysis of genomic data and meiocyte expression patterns and/or reproductive structures in other plants, as well as the completion of the sunflower genome and advances in functional genomic tools in this plant.

## Methods

### Meiocyte collection, RNA extraction, and sequencing

Sunflower plants of three genotypes: domesticated (inbred line HA89, *Helianthus annus* L. *var. macrocarpus*), F1 (F1 generation cross between domesticated and wild genotypes) and wild (Ac-8, *Helianthus annuus* L. *ssp. texanus*), were grown under greenhouse conditions as previously described [17]. At the beginning of the R2 development stage [81], approximately 10 disc florets of the floral bud were squashed with dissecting needles on a concave glass slide with 80 μL sterile distilled water. A first filter to confirm the meiotic stage was performed under a microscope (without staining) to determine whether the meiocytes remained associated to form the characteristic “worm” structure of prophase I meiocytes [15]. If the meiocytes appeared to be in early meiotic stages and pollen grains or tetrads were absent, a subsample of the disc floret was fixed in a 96 % ethanol:acetic acid solution (3:1) for 24 h, and then observed under a microscope with the squashed-acetocarmine staining method to confirm the meiotic phase. Once samples passed this ‘double-check’ protocol, meiocytes were collected from developmentally-matched florets in RNAlater (Ambion, Inc.) and stored at −70 °C until RNA extraction.

RNA of prophase I meiocytes from each genotype was isolated using the ZR RNA MicroPrep kit (Zymo Research, Orange, CA) following the manufacturer’s instructions, and stored at −70 °C. Six libraries from meiocytes (two biological replicates for each genotype) were prepared using standard Illumina TruSeq RNA library preparation kits, and sequenced using the Illumina HiSeq 2500 platform to obtain 100 bp paired-end reads.

### Quality filtering, *de novo* assembly, and gene expression estimation

Adaptors were removed from the reads using cutadapt 1.3 software [82]. Adaptor-free reads were subsequently quality-trimmed using PRINSEQ 0.20.4 software [83], allowing a minimum quality score of 20 and no more than two ambiguous bases per read. Then, *de novo* assembly of the trimmed reads was performed using Trinity (release 20140413) software [84] and default parameters. The assembler classifies the output in two categories: ‘genes’, corresponding to sequences that the algorithm considers are the product of different genes, and ‘transcripts’, which contain sequences with slight differences between them so as to be cataloged as a different gene; these could be splice variants or distinct alleles. Thus, to quantify the expression, we selected the longest sequence of each reconstructed ‘gene’ and remapped the reads using Bowtie2 2.1.0 (set-up parameters: −a –rdg 6, 5 –rfg 6, 5 –score-min L, −0.6, −0.4) [85]. Those reads that mapped exclusively to one gene (unique read counts) were estimated using eXpress 1.4.1 [86] with default parameters. These counts were arranged in a matrix for subsequent analysis.

### Gene identification

Transcripts from protein coding genes present in the assembled transcriptome were identified by sequentially querying them to four different peptide databases using blastx [87], which translates the six reading frames of the transcript to the corresponding peptide sequences, and then look for significant similarities with the peptide database. Hits were considered significant if the bit-score of the alignment was ≥90 and had an expected value E ≤ 10^−6^. The use of a threshold of bit-score ≥90 guarantees a minimum average alignment of approximately 100 aa (300 bp), and thus is very likely to correctly identify the peptide coded by a transcript. First, transcripts were compared against *A. thaliana* (TAIR10) peptides, and transcripts that passed the above threshold where considered as protein-coding and identified by the corresponding *A. thaliana* ortholog. Transcripts without a significant hit where compared, in turn, with sunflower peptide dataset for varieties HA412 and HAXRQ, available at the HeliaOrg website (https://www.heliagene.org) and finally with NCBI RefSeq plant peptides (release 24/07/2013). At each step sequences having a significant hit (bit-score ≥90 and E ≤ 10^−6^) were considered as protein coding and identified with the corresponding peptide, and only the ones without a significant hit were used in the next steps. We used first TAIR10 given that *A. thaliana* has the best curated set of plant genes and proteins. Transcripts without a significant hit to TAIR10 were compared to sunflower peptide databases to account for peptides specific to this genus and with no ortholog in *A. thaliana,* while comparison with RefSeq plant peptides covered the possibility of peptides missed from the previous databases. Genes having no hits among any of the queried peptide databases were cataloged as ‘unidentified’ and used for subsequent lncRNA identification analyses. A MySQL relational database (Server version 5.5.34) that included all data from assembly, mapping, and annotation was also compiled.

### Identification of lncRNA

Since all unidentified transcripts had a length >200 nt, length filtering was not necessary. Two different algorithms were used to determine if a transcript could be the product of a lncRNA: a coding protein calculator analysis tool in the CPC web interface (http://cpc.cbi.pku.edu.cn) [36] and analysis with the Coding-Potential Assessment Tool (CPAT) [37]; default parameters were used for both. For the CPAT analysis, the protein coding gene models were constructed using the *A. thaliana* genome (TAIR10). Only genes that passed the threshold for both analyses (CPC score ≤ −1 and CPAT score ≤ 0.3) were cataloged as lncRNAs. Even when the nominal threshold for CPAT score was set to ≤ 0.3, all the lncRNAs reported here have a very low value of protein coding probability, CPAT score ≤ 1 × 10^−6^ (Table AF1-4 in Additional file 1), and the simultaneous application of the two algorithms excluded 7479 (21.80 %) sequences as putative lncRNA (Table AF1-3 in Additional file 1). We also show that our criteria to catalogue genes as lncRNAs are at least as stringent as the ones used in 17 recent references mentioning lncRNA detection (see Table AF1-5 and section “Additional discussion of lncRNA Identification” in Additional file 1). We also did a blastn analysis of the identified lncRNAs with the genome draft assembly of the sunflower inbred line HA412 (Celera_14libs_sspace2_ext.final.scaffolds.split.fasta) deposited in the Sunflower Genome Project website: http://www.sunflowergenome.org, to which Prof. Loren Rieseberg (University of British Columbia) kindly gave us access. A hit with the genome was considered significant if the result had a bitscore ≥90 and an expected value E ≤ 10^−6^.

### Identification of putative targets for lncRNAs

Software package ‘LncTar’ [76] (version of September-01, 2015) was downloaded from site http://www.cuilab.cn/lnctar, and run in a subset of the sunflower lncRNAs and meiotic genes. Details of the procedure and results are presented in section “Putative targets for lncRNAs” of Additional file 1.

### Small RNA sequencing and mapping

RNA from prophase I meiocytes from domesticated and wild genotypes was isolated using the ZR RNA MicroPrep kit (Zymo Research, Orange, CA) following the manufacturer’s instructions and modifications for sRNAs extractions, and stored at −70 °C. Two libraries (one for each genotype) were prepared using standard Illumina TruSeq Small RNA library preparation kits, and sequenced using the Illumina HiSeq 2500 platform to obtain 37 bp single-end reads. Reads were quality-trimmed using the Kraken set of tools [88], and those reads with a length between 20 and 25 nt were selected for subsequent analyses. Mapping of the sRNA reads was performed using the Bowtie 1.1.2 version [89] to a mixed reference of the genome draft contigs (described above) and meiocyte transcriptome assembly from this study with the following parameters: −v 1 --best --strata -a -f --chunkmbs 512.

### Description of lncRNA characteristics: NATs and Transposons (TEs)

To identify lncRNAs that were natural antisense transcripts (NATs), we downloaded the sequences of the predicted *Helianthus annus* natural antisense transcripts from the PlantNATsDB (http://bis.zju.edu.cn/pnatdb/) [90], and queried our lncRNAs against them using blastn. To identify TEs and repetitive sequences in the lncRNAs, we did a blastn search against sequences in the sunflower repetitive sequences database SUNREP [71], RepBase (Version 21.02) [91] and PGSB Repeat Element Database (PGSB-REdat) [92]. BLAST cutoff values were the same for both analyses: bit-score ≥70 and expected value E ≤ 10^−6^. Additionally, we conducted a search of repetitive elements in our lncRNAs by using the Web Server tool of RepeatMasker Version open-4.0.5 (http://www.repeatmasker.org/cgi-bin/WEBRepeatMasker) (A.F.A. Smit, R. Hubley and P. Green, unpublished), with the ‘slow’ option, in order to have the maximum sensitivity.

### Statistical design and analyses

To perform differential expression analyses, we did two biological replicates for each sunflower genotype with two different sets of plants (floral buds, florets), and performed independent RNA isolation, library construction, and sequencing. Expression variations found for each gene between the two biological replicates gave an estimate of the statistical error (unexplained variation), which includes biological as well as technical variation. As previously described, the gene expression level was considered as the unique read counts obtained for each gene. However, for genes sharing the same peptide identifier, expression data were collapsed to a single gene by summing the numbers of reads that mapped to each component. We collapsed the counts according to their identifier in the following order: TAIR10, HA412, HAXRQ, and Refseq. To measure the number of missing genes in our transcriptome and to gauge completeness, we used the method described by García-Ortega and Martínez [35]. Differential expression analysis was made with the edgeR package [93], and the resulting p-values were input into the q-value function [94] with default parameters, setting the fdr.level = 0.01 to obtain a FDR of 1 %. Differences in proportions were assessed with a two-tailed test for population proportion and a threshold of *P* < 0.01. All statistical analyses were conducted in R version 2.15.3 [95].

## Additional file

Additional file 1: Additional text, tables and figures. Text, Tables and Figures from this file are referred as “AF1-#”, where “#” is the corresponding number in both, the main text and Additional file 1. (PDF 1223 kb)
