# Supplementary material for: Long non-coding RNAs are major contributors to transcriptome changes in sunflower meiocytes with different recombination rates
Source: BMC Genomics. 2016 Jul 11;17:490. doi: 10.1186/s12864-016-2776-1 (PMC4940957; doi:10.1186/s12864-016-2776-1)
Supplement: Additional file 1: — Additional text, tables and figures. Text, Tables and Figures from this file are referred as “AF1-#”, where “#” is the corresponding number in both, the main text and Additional file 1. (PDF 1223 kb) [file 12864_2016_2776_MOESM1_ESM.pdf]

# - Additional File 1 -
Additional Text, Tables and Figures for "Long non-coding RNAs are major contributors to transcriptome changes in sunflower meiocytes with different recombination rates"

NATHALIA M.V. FLÓREZ-ZAPATA, M. HUMBERTO REYES-VALDÉS AND OCTAVIO MARTÍNEZ*
*Corresponding author omartine@langebio.cinvestav.mx, octavio.martinez@cinvestav.mx

Here we present additional text (details of methods and discussion), tables and figures. Sections of this document are refered in the main text, and numbers of tables and figures are referred in the main text as "AF1-#", where "#" is the corresponding number for table or figure. Text in blue includes link to web resources.

Contents

Sequencing and assembly results 1
Additional discussion of lncRNA Identification 2
Putative targets for lncRNAs 4
Supplementary Figures 10
Transposons (TEs) and Repetitive Elements (REs) in sunflower lncRNAs 14
qRT-PCR analysis of selected lncRNAs 16
References 19

Sequencing and assembly results

We used the assembly from F_1 meiocytes as a representative transcriptome for differential expression analysis since alleles from both parents are expected to be present and expressed in the hybrid. 73,669 transcripts (with a N50=1,298) composed this assembly, of these, 73,658 showed expression in at least one of the genotypes. From this transcriptome, we identified 71 genes orthologs to *Arabidopsis thaliana* known meiotic genes, 8 more than previously described in a transcriptome assembled de novo from meiocytes and somatic sequences [5]. This confirmed the reliability and representativeness of the F_1 transcriptome. We observed that when we assembled the transcriptome with sequences from the F_1 and one of the parents, the complexity of the transcriptome increased, i.e., more genes as well as splice variants were reconstructed, but the number of known-meiotic genes detected was the same. So we thought that the possibilities of miss-assemblies and chimeric transcripts increases when reads from different genotypes are mixed, but not new genes are detected.

On the other hand, after testing for the number of missing genes in this transcriptome through the methodology described in [6], we conclude that the F_1 transcriptome is complete. Table AF1-1 presents the number of reads obtained from each one of the libraries, as well as the number and percentages of reads with unique hit to the F_1 assembly. The uniformity of the percentages of reads from each library mapping to the F_1 transcriptome demonstrate that no significant bias in expression was introduced by using the F_1 transcriptome.

In Table AF1-1 we can see that the percentages of reads that have a unique hit with the F_1 transcriptome for meyocites goes from a minimum of 75.26 up to a maximum of 79.91, with an average of 77.90, a median of 78.40 and a standard deviation of only 1.86%. The number and percentages of reads from the somatic transcriptome previously obtained in [5] were used to compare the lncRNA detected in the somatic and meyocites transcriptomes.

Date: May 5, 2016.

Table AF1-1. **Number of paired reads obtained and uniquely mapped to the F_1 assembly per library.** Biological replicates for each sample are represented as BR, percentages of mapped reads (%) are estimated with reference to the total number of reads in each library.

		Meiocytes	
Library	Replicate	Total Reads	Mapped Reads (%)
Domesticated	BR1	179,789,421	139,532,735 (**77.61**%)
	BR2	142,454,268	112,861,139 (**79.23**%)
F_1	BR1	189,180,804	151,174,022 (**79.91**%)
	BR2	93,914,482	74,366,257 (**79.19**%)
Wild	BR1	123,666,567	94,260,868 (**76.22**%)
	BR2	132,921,292	100,034,300 (**75.26**%)
	Total	861,926,834	672,229,321 (**77.99**%)
Somatic transcriptome (previously obtained)			
Library	Replicate	Total Reads	Mapped Reads
Somatic	R1	173,458,030	128,984,174 (**74.36**%)

Table AF1-2 present the sequencing results for the sRNA libraries in the wild and domesticated genotypes.

Table AF1-2. Number of clean reads obtained in the sequencing of sRNA transcriptomes of prophase I meiocytes from wild and domesticated sunflower genotypes.

Length	Wild genotype		Domesticated genotype	
20	87,931	(1.66%)	164,495	(3.11%)
21	452,676	(8.57%)	678,774	(12.85%)
22	408,185	(7.73%)	420,184	(7.96%)
23	729,207	(13.81%)	803,569	(15.22%)
24	3,425,818	(64.87%)	3,042,461	(57.61%)
25	177,383	(3.36%)	171,924	(3.26%)
Total	5,281,200	(100.00%)	5,281,407	(100.00%)

Additional discussion of lncRNA Identification

'Coding protein calculator' (CPC) [11] and 'Coding-Potential Assessment Tool' (CPAT) [28] were employed conjointly to assure a high confidence assignment of transcripts as lncRNA. These two algorithms are complementary; CPC assesses protein-coding potential of transcripts using sequence features and support vector machine, while CPAT uses an alignment-free logistic regression model. For CPC, negative scores indicate low coding potential, and a maximum threshold of -1 is considered as strong evidence of non-coding potential, and thus in our case as authentication for lncRNAs. On the other hand, CPAT directly gives an estimate of the probability that the sequence is coding for a protein, allowing a direct interpretation of the results. Here we give details of the selection process and compare it with the use of the two algorithms in the literature.

All 34,304 sunflower genes without a good blastx hit to proteins were subjected to the CPC and CPAT algorithms. For the classification of transcripts as lncRNAs we set a double threshold, considering a gene as lncRNA only for transcripts for which CPC score ≤ -1 and CPAT score ≤ 0.3 Table AF1-3 presents the number and percentages of sequences which fulfilled each criterion.

In Table AF1-3 we can appreciate how the application of both criteria for the selection of lncRNA was successful in avoiding putative false positives, that will happen if only the criterion CPC ≤ -1 but not

Table AF1-3. Number and percentages of sequences fulfilling the CPC and CPAT score thresholds.

CPC ≤ -1?	CPAT≤ 0.3?	Genes	Percentage
No	No	1,498	4.37
No	Yes	2,714	7.90
Yes	No	4,765	13.89
Yes	Yes	25,327	73.83
	Total:	34,304	100.00

CPAT ≤ 0.3 or vice versa are applied; on those cases 4,765 (13.89%) or 2,714 (7.90%) of the sequences will be called as lncRNA. Thus, in total the double filtering eliminated lncRNA 4,765 + 2,714 = 7,479 (21.80%) as putative lncRNA, letting only the 25,327 sequences reported (fourth row of Table AF1-3). Table AF1-4 presents the minimum, average and maximum of the scores for genes classified as lncRNA and "Unknown".

Table AF1-4. Statistics for CPC and CPAT scores in two groups of transcripts. Values for the minimum (Min.), average (Avg.) and maximum (Max.) are presented for the groups of genes finally classified as lncRNA or "Unknown" (Unclassified).

			CPC			CPAT	
Group	Number	Min.	Ave.	Max.	Min.	Avg.	Max.
lncRNA	25,327	-1.61	-1.26	-1.00	2×10^{-6}	2×10^{-6}	2×10^{-6}
Unknown	8,977	-1.57	-1.01	2.74	2.22	0.70	1.00
Total:	34,304	-1.61	-1.20	2.74	2×10^{-6}	0.18	1.00

From Table AF1-4 we can see that the average CPC score for genes classified as lncRNA is -1.26, while for the group classified as 'Unknown' this average is -1.01, but has a maximum of 2.47, much larger than the threshold of -1.00 set for lncRNAs, thus CPC was an effective discriminant criteria. However, the CPAT threshold of a score less or equal than 0.3 in coding probability, resulted even more powerful to determine non-coding capacity; while nominally we asked the value to be equal or less than 0.3, effectively all lncRNAs detected have the same value of in coding probability, 2×10^{-6}, indicating a near null possibility for these sequence to be coding protein. Thus, *a posteriori*, the maximum value of the CPAT parameter for our lncRNA sequences is far from 0.3 and very near zero.

To compare the stringency of the thresholds set to CPC and CPAT in our procedure to designate lncRNAs, we compiled Table AF1-5 showing references that use these algorithms in distinct organisms.

From Table AF1-5 we can see that in references [12, 31, 27, 33, 34, 19] the threshold set to CPC, 0, is less stringent the value set by us, -1, while in references [17, 16, 8, 10, 29] this threshold is equally stringent than the one set by us. On references [26, 1, 2, 22, 32], the threshold for the CPAT algorithm is more permissive than the one set by us, i.e., in all cases > 0.3 More relevant, in all references presented in Table AF1-5, except by [30], only a single algorithm is employed to call lncRNA, while we employed thresholds for both programs, and as we have demonstrated (see Table AF1-3), this fact filter a large number of putative false positive lncRNAs. Only in [30] both programs are employed conjointly to call lncRNA, and in that case our threshold for CPC is more stringent, ≤ -1, than the one in [30], ≤ 0.5, while the threshold for CPAT in [30] is 0.02, nominally more stringent that the threshold used by us, 0.3; however, as shown in Table AF1-4 in all our 25,327 lncRNAs the CPAT value is near zero (2×10^{-6}) and thus our classification results are at least as robust than the ones presented in [30].

In summary, we have shown that our results of classification of the 25,327 as lncRNAs are at least as robust, but likely more trustworthy than the ones presented in the 17 references, corresponding to 17 different organisms, presented in Table AF1-5.

Table AF1-5. **Review of thresholds considered for lncRNA identification through CPC and CPAT algorithms.** Genes with a score less or equal than the one presented were considered as lncRNA in the corresponding references.

Row	Algorithm	Organism	Threshold Score (≤)	Reference
1	CPC	*Ganoderma lucidum*	0	[12]
2	CPC	*Drosophila melanogaster*	0	[31]
3	CPC	*Solanum lycopersicum*	0	[27]
4	CPC	*Oryza sativa*	0	[33]
5	CPC	*Gossypium arboreum*	0	[34]
6	CPC	*Musa spp*	0	[19]
7	CPC	*Mus musculus*	-1	[17, 16]
8	CPC	*Apis cerana & mellifera*	-1	[8]
9	CPC	*Danio rerio*	-1	[10]
10	CPC	*Medicago truncatula*	-1	[29]
11	CPAT	*Tripterygion delaisi*	0.500	[26]
12	CPAT	Bovine (Limousin bull)	0.348	[1]
13	CPAT	*Mus musculus*	0.440	[2]
14	CPAT	Human	0.364	[2]
15	CPAT	*Anopheles gambiae*	0.390	[22]
16	CPAT	*Arabidopsis thaliana*	0.365	[32]
17	CPC	Bovine	0.500	[30]
18	CPAT	Bovine	0.020	[30]

Putative targets for lncRNAs

As shown in multiple model systems, lncRNAs can form networks of ribonucleoprotein (RNP) complexes with chromatin regulators, and thus can function as scaffolds in these complexes [23]. With the aim of predicting putative targets (protein coding genes) for the sunflower lncRNA found in this study, we tested the 'LncTar' algorithm [13] with a subset of our data.

The method implemented in LncTar [13] assume that base pairing plays a critical role in RNA-RNA interactions, and works by estimating the approximate binding free energy, delta-G (dG) for a pair of sequences. The value of dG is then normalized by dividing it by the minimum of the length of the two sequences, obtaining normalized delta-G (ndG). Values of ndG which are more negative indicate a higher probability of 'interaction' between the sequences, even when such interactions do not follow the canonical rules of base-pairing, and are calculated by Nearest-Neighbor doublets. Pairs of sequences that surpass a given threshold are reported by the program. In [13] authors test their method with 10 pairs of corroborated lncRNA / mRNA sequences, successfully predicting 8 of the 10 pairs by using a ndG threshold ndG< -0.1 The manual of the program suggests cutoffs for ndG -0.08 (low confidence), -0.10 (low confidence), -0.13 (medium confidence), -0.15 (high confidence), and -0.20 (very high confidence).

It is important to notice that the authors in [13] do not clarify the proportion of false positives that their method is likely to give. They tested 5000 pairs of random lncRNA / mRNAs, and found that the threshold of ndG=-0.10 is surpassed (ndG < -0.1) by around 5% of the random pairs; this implies that with that threshold 5% of the 'significant' pairs will be false positives, even if 8 of 10 experimentally confirmed interactions were recovered. To take a much higher threshold for ndG is not a good solution, given that as discussed in [13], true interaction are dependent on other factors, as stacked pair energy, loop energy and RNA tertiary structure are not taken into account. Thus, any interaction found with this method demands experimental confirmation to demonstrate the putative targeting of a gene by a lncRNA.

LncTa was downloaded from http://www.cuilab.cn/lnctar and installed to perform the analyses. A test run showed that in our computer system the program takes around 4.5 seconds to process a single pair of sequences. Given that we have a total of 59,085 genes, of which 33,758 are protein coding and 25,327 are lncRNAs, the number of comparison of all possible pairs of protein coding and lncRNAs to find putative targets is very large, $33,758 \times 25,327 = 854,988,866$, and thus it is unfeasible to perform all these comparisons in a reasonable time, even if the computation time is reduced by the use of a more powerful computer or distributed in a computer cluster.

To make a test run with '`LncTar`' we decided to use a subset of the previously described meiotic genes found in the domesticated genotype [5]. From these genes we selected the 10 with largest differences in expression between the parental genotypes.

Reasoning that these 10 DE meiotic genes could be targets of lncRNAs that were also DE between parental genotypes, we selected 473 lncRNAs which were DE between parents with a Q-value $\leq 1e-20$. With these two sets of sequences we have $10 \times 473 = 4730$ pair comparisons to do. `LncTar` was run with these sets and resulted in the detection of 12 pairs lncRNA / mRNA (mRNA are the transcripts of the meiotic genes) with a ndG < -0.15. Table AF1-6 presents the pairs of lncRNA / putative meiotic targets found in the run.

Table AF1-6. Putative targets found for 12 lncRNAs in a set of differentially expressed meiotic genes

Row	lncRNA	Putative Target	ndG	Gene function
1	lncRNA-c24788_g2_i1	c53699_g4_i1-AESP	-0.68	Sister Chromatid cohesion
2	lncRNA-c57649_g1_i1	c53699_g4_i1-AESP	-0.22	Sister Chromatid cohesion
3	lncRNA-c23889_g1_i1	c53699_g4_i1-AESP	-0.21	Sister Chromatid cohesion
4	lncRNA-c43609_g2_i1	c45048_g1_i1-MMD1	-0.32	Cell cycle: spindle cytokinesis
5	lncRNA-c66209_g1_i1	c45048_g1_i1-MMD1	-0.16	Cell cycle: spindle cytokinesis
6	lncRNA-c24499_g2_i1	c45048_g1_i1-MMD1	-0.16	Cell cycle: spindle cytokinesis
7	lncRNA-c49554_g1_i1	c52764_g1_i3-MSH5	-0.17	Recombination: CO and NCO outcome
8	lncRNA-c18889_g2_i1	c52764_g1_i3-MSH5	-0.16	Recombination: CO and NCO outcome
9	lncRNA-c38193_g2_i1	c50732_g1_i1-RAD51	-0.17	Recombination: early DSB repair
10	lncRNA-c32101_g1_i1	c43375_g1_i1-XRCC3	-0.22	Recombination: early DSB repair
11	lncRNA-c17335_g1_i1	c26076_g1_i1-ZIP4	-0.27	Recombination: CO and NCO outcome
12	lncRNA-c27794_g1_i1	c26076_g1_i1-ZIP4	-0.16	Recombination: CO and NCO outcome

From Table AF1-6 we observe that 12 lncRNAs have as putative targets 6 sunflower meiotic genes. In two cases, 3 distinct lncRNAs have the same target (genes AESP and MMD; rows 1 to 3 and 4 to 6, respectively); in two cases two distinct lncRNAs have the same targets (genes MSH5 and ZIP4; rows 7 to 8 and 11 to 12, respectively) while in the remaining two cases (genes RAD51 and XRCC3; rows 9 and 10, respectively) the relation is one to one (lncRNA to target). Note that even if we use the most stringent threshold for ndG quoted in the software manual, -0.20 ('very high confidence'), only rows 5 to 9 and 12 in Table AF1-6 will be eliminated, letting 6 interactions, i.e., 4 meiotic genes with putatively targeted by 6 lncRNAs.

It is also interesting to see if there is any correlation between the expression of the lncRNA and their putative targets. Expression levels in Transcripts per Million (TPM) and correlations between expression levels in the three genotypes are shown in Table AF1-7.

From Table AF1-7 we can see that the expression levels of the meiotic genes is, as expected, much larger than the one for the lncRNA. This was in general the case in all our data; lncRNAs presented a much smaller relative expression than the protein coding genes. Also from From Table AF1-7 we can observe that for some of the pairs 'lncRNA / putative target' there is a high correlation in expression level when measured in the three genotypes (D=Domesticated, F_1 and Wild); rows 4 to 10 and 12 (8/12) have

Table AF1-7. Expression levels per genotype and estimated correlation ($\hat{r}$) between expression levels of lncRNAs and their putative targets.

			Estimated expression (TPM)						
			Putative Target			lncRNA			
Row	lncRNA	Putative Target	D	F_1	W	D	F_1	W	$\hat{r}$
1	lncRNA-c24788_g2_i1	c53699_g4_i1-AESP	20	23	6	0.00	0.6	1.84	-0.8774
2	lncRNA-c57649_g1_i1	c53699_g4_i1-AESP	20	23	6	0.00	3.52	2.08	0.0703
3	lncRNA-c23889_g1_i1	c53699_g4_i1-AESP	20	23	6	1.91	0.81	0.01	0.7038
4	lncRNA-c43609_g2_i1	c45048_g1_i1-MMD1	34	55	182	0.00	3.15	15.38	0.9983
5	lncRNA-c66209_g1_i1	c45048_g1_i1-MMD1	34	55	182	0.00	0.33	3.3	0.9990
6	lncRNA-c24499_g2_i1	c45048_g1_i1-MMD1	34	55	182	0.00	0.72	2.41	0.9875
7	lncRNA-c49554_g1_i1	c52764_g1_i3-MSH5	33	52	124	4.14	2.48	0.04	0.9987
8	lncRNA-c18889_g2_i1	c52764_g1_i3-MSH5	33	52	124	0.00	1.66	3.8	-0.9775
9	lncRNA-c38193_g2_i1	c50732_g1_i1-RAD51	29	43	133	0.00	0.51	2.68	0.9692
10	lncRNA-c32101_g1_i1	c43375_g1_i1-XRCC3	3	2	1	2.38	13.37	57.09	-0.9939
11	lncRNA-c17335_g1_i1	c26076_g1_i1-ZIP4	26	53	71	0.00	0.18	1.71	0.8596
12	lncRNA-c27794_g1_i1	c26076_g1_i1-ZIP4	26	53	71	0.00	0.76	1.06	0.9905

an absolute value of $\hat{r} > 0.9$ Figure AF1-2 present the plot of relative expression by genotype for the case of the meiotic gene c45048_g1_i1-MMD1 which was identified as putative target for the lncRNAs lncRNA-c43609_g2_i1, lncRNA-c66209_g1_i1 and lncRNA-c24499_g2_i1 (rows 4 to 6 in tables AF1-6 and AF1-7).

From Figure AF1-1 we can see that the tendency for expression change between the meiotic gene c45048_g1_i1-MMD1 and the three lncRNAs putatively targeting it (lncRNAs lncRNA-c43609_g2_i1, lncRNA-c66209_g1_i1 and lncRNA-c24499_g2_i1) is very alike; for the four genes the lowest expression is found at the domesticated genotype (D), increasing in F_1 and then given the maximum for the wild genotype (W). The concordance of the expression pattern gives as result the very high correlations ($\hat{r} > 0.9874$) between the expression patterns (see rows 4 to 6 in Table AF1-7).

To avoid jumping to conclusions about the reliability of the interactions found, we designed a 'control group' of meiotic genes. These were the 10 meiotic genes with the smallest evidence of differential expression (less significant, $P > 0.8$) between genotypes. These not-DE meiotic genes were run as putative targets of the set of lncRNAs previously selected. From this run a set of 20 significant (ndG$< -.15$) interactions between lncRNA and non-differentially expressed meiotic genes were found. Names of genes ndG and gene function are presented in Table AF1-8.

Table AF1-8 shows the 20 interactions found between 18 distinct lncRNA (note that lncRNA-c58535_g1_i1 is present in rows 2, 15 and 20) and 7 distinct not-differentially expressed meiotic genes. All the lncRNAs shown in Table AF1-8 are distinct to the ones previously found (tables AF1-6 and AF1-7).

Table AF1-9 shows the expression levels per genotype and estimated correlation ($\hat{r}$) between expression levels of lncRNAs and their putative targets (meiotic genes not-differentially expressed).

From Table AF1-9 we can observe that, even when the control set of non-differentially expressed meiotic genes presents relatively homogeneous expression in the three genotypes, some of the absolute values of correlation estimated with the expression in the lncRNAs are large, $\hat{r} > 0.9$ (rows 1, 2 and 4) even when in general the correlations are smaller than with the set of differentially expressed meiotic genes (Table AF1-6).

The logic for the selection of a control group of not-differentially expressed meiotic genes was that given that these genes do not vary in the genotypes, they were unlikely to be controled by lncRNA which are differentially expressed in the same genotypes. However, the number of 'significant' interactions in the 'control' group (tables AF1-8 and AF1-9) is larger, 20 interactions, than the ones detected with the set

Table AF1-8. Putative targets found for 18 lncRNAs in a control set of non-differentially expressed meiotic genes

Row	lncRNA	Putative Target	ndG	Gene function
1	lncRNA-c7029_g1_i1	c34014_g1_i1-AML1	-0.21	Entry into meiosis
2	lncRNA-c58535_g1_i1	c34014_g1_i1-AML1	-0.20	Entry into meiosis
3	lncRNA-c28777_g1_i2	c34014_g1_i1-AML1	-0.19	Entry into meiosis
4	lncRNA-c41972_g2_i2	c34014_g1_i1-AML1	-0.18	Entry into meiosis
5	lncRNA-c34814_g1_i2	c34014_g1_i1-AML1	-0.18	Entry into meiosis
6	lncRNA-c24881_g1_i1	c36374_g1_i1-AML4	-0.19	Entry into meiosis
7	lncRNA-c69494_g1_i1	c23367_g1_i1-ASK2	-0.87	Cell cycle: spindle cytokinesis
8	lncRNA-c62935_g1_i1	c23367_g1_i1-ASK2	-0.22	Cell cycle: spindle cytokinesis
9	lncRNA-c60729_g1_i1	c23367_g1_i1-ASK2	-0.20	Cell cycle: spindle cytokinesis
10	lncRNA-c30556_g1_i1	c23367_g1_i1-ASK2	-0.17	Cell cycle: spindle cytokinesis
11	lncRNA-c27496_g1_i1	c23367_g1_i1-ASK2	-0.16	Cell cycle: spindle cytokinesis
12	lncRNA-c53816_g4_i1	c45617_g1_i1-MAD2	-0.52	Cell cycle: spindle cytokinesis
13	lncRNA-c48589_g5_i1	c51354_g1_i1-RAD50	-0.18	Recombination: early DSB repair
14	lncRNA-c44658_g2_i1	c20232_g2_i1-RBR	-0.25	Recombination: CO and NCO outcome
15	lncRNA-c58535_g1_i1	c20232_g2_i1-RBR	-0.21	Recombination: CO and NCO outcome
16	lncRNA-c53532_g3_i2	c20232_g2_i1-RBR	-0.21	Recombination: CO and NCO outcome
17	lncRNA-c29570_g1_i1	c20232_g2_i1-RBR	-0.19	Recombination: CO and NCO outcome
18	lncRNA-c65312_g1_i1	c20232_g2_i1-RBR	-0.18	Recombination: CO and NCO outcome
19	lncRNA-c50081_g3_i1	c51468_g1_i2-RFC1	-0.21	Recombination: CO and NCO outcome
20	lncRNA-c58535_g1_i1	c51468_g1_i2-RFC1	-0.18	Recombination: CO and NCO outcome

Table AF1-9. Expression levels per genotype and estimated correlation ($\hat{r}$) between expression levels of lncRNAs and their putative targets in a control set of non-differentially expressed meiotic genes.

			Estimated expression (TPM)						
			Putative Target			lncRNA			
Row	lncRNA	Putative Target	D	F_1	W	D	F_1	W	$\hat{r}$
1	lncRNA-c7029_g1_i1	c34014_g1_i1-AML1	117	115	111	0.00	0.55	3.02	-0.9978
2	lncRNA-c58535_g1_i1	c34014_g1_i1-AML1	117	115	111	2.75	1.30	0.02	0.9498
3	lncRNA-c28777_g1_i2	c34014_g1_i1-AML1	117	115	111	0.00	2.67	2.42	-0.6290
4	lncRNA-c41972_g2_i2	c34014_g1_i1-AML1	117	115	111	2.16	1.42	0.03	0.9943
5	lncRNA-c34814_g1_i2	c34014_g1_i1-AML1	117	115	111	0.00	2.78	2.46	-0.6105
6	lncRNA-c24881_g1_i1	c36374_g1_i1-AML4	46	52	45	0.27	11.92	29.94	-0.1781
7	lncRNA-c69494_g1_i1	c23367_g1_i1-ASK2	832	708	845	0.00	3.88	3.21	-0.5683
8	lncRNA-c62935_g1_i1	c23367_g1_i1-ASK2	832	708	845	0.00	1.68	6.95	0.3624
9	lncRNA-c60729_g1_i1	c23367_g1_i1-ASK2	832	708	845	0.00	0.36	0.95	0.2209
10	lncRNA-c30556_g1_i1	c23367_g1_i1-ASK2	832	708	845	0.00	0.41	2.40	0.4312
11	lncRNA-c27496_g1_i1	c23367_g1_i1-ASK2	832	708	845	0.02	2.70	5.70	0.1143
12	lncRNA-c53816_g4_i1	c45617_g1_i1-MAD2	52	43	53	1.32	0.56	0.00	-0.0356
13	lncRNA-c48589_g5_i1	c51354_g1_i1-RAD50	58	77	56	2.21	0.82	0.01	-0.0760
14	lncRNA-c44658_g2_i1	c20232_g2_i1-RBR	117	123	118	0.00	0.12	1.63	-0.3176
15	lncRNA-c58535_g1_i1	c20232_g2_i1-RBR	117	123	118	2.75	1.3	0.02	-0.1679
16	lncRNA-c53532_g3_i2	c20232_g2_i1-RBR	117	123	118	2.82	1.46	0.03	-0.1167
17	lncRNA-c29570_g1_i1	c20232_g2_i1-RBR	117	123	118	0.00	1.37	2.48	0.1946
18	lncRNA-c65312_g1_i1	c20232_g2_i1-RBR	117	123	118	0.00	3.30	4.78	0.3438
19	lncRNA-c50081_g3_i1	c51468_g1_i2-RFC1	101	96	100	2.59	0.91	0.00	0.2195
20	lncRNA-c58535_g1_i1	c51468_g1_i2-RFC1	101	96	100	2.75	1.30	0.02	0.0875

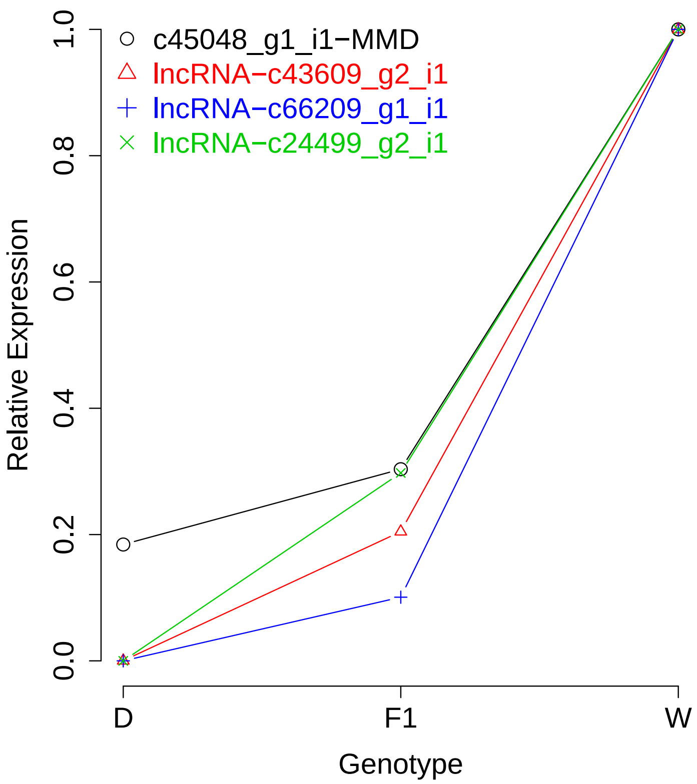

Figure AF1-1. Plot of gene expression relative to the one obtained in the wild (W) genotype for the meiotic gene c45048_g1_i1-MMD1 which was identified as putative target for the lncRNAs lncRNA-c43609_g2_i1, lncRNA-c66209_g1_i1 and lncRNA-c24499_g2_i1 (rows 4 to 6 in tables AF1-6 and AF1-7).

of differentially expressed genes (tables AF1-6 and AF1-7), 12 interactions. Even when it cannot be ruled out the some of the interactions found with the 'control' group could be legitimate, it results highly suspicious that more interactions are found where it was expected to find less.

To make a further test of the reliability of the algorithm, we defined a second control group formed by 10 genes with very large expression in the somatic transcriptome but very low expression in meiocytes. The rational for this last experiment is that genes with very low expression in meiocytes, but high expression in the somatic tissues are unlikely to be control by lncRNA differentially expressed in meiocytes. However, this second control experiment produced 49 interactions with a ndG < -0.15. Table AF1-10 summarize statistics for ndG in the three experiments carried out to detect interactions between the set of 473 differentially expressed lncRNAs and the 'Test' set of 10 meiotic differentially expressed genes (tables AF1-6 and AF1-7), the 'Control 1', consisting on 10 meiotic not-differentially expressed genes (tables AF1-8 and AF1-9) and finally 'Control 2', the set of 10 (non meiotic) protein coding genes strongly expressed in somatic tissues but not in meiocytes.

From Table AF1-10 we can see that the number of 'significant' interactions detected (n) is larger in the two control groups ('Control 1' and 'Control 2' rows) than in the original test ('Test' row), demonstrating that very likely many, if not all, of the detected interactions could be biologically irrelevant. It cannot be ruled out that some of the interactions detected could be true interactions between lncRNAs and putative targets, however, there is clearly a large number of interactions that are false positives, otherwise the number of interactions detected in the Test group will be larger, or at least equal, to the number of interactions in the control groups, but the opposite happens. Even with the relatively small number of interactions explored, $470 \times 10 = 4730$, for the groups of 470 lncRNAs and 10 protein coding genes at each case, $n = 12, 20, 49$ interactions are detected for 'Test', 'Control 1' and 'Control 2' respectively. Also

Table AF1-10. **Statistics for ndG in the three groups of genes searched.** Test - 10 meiotic differentially expressed genes (tables AF1-6 and AF1-7); Control 1 - 10 meiotic not-differentially expressed genes (tables AF1-8 and AF1-9); Control 2 - 10 protein coding genes strongly expressed in somatic tissues but not meiocytes.

Group	n	Min.	1st Qu.	Median	Mean	3rd Qu.	Max.	S
Test	12	-0.675	-0.2357	-0.1884	-0.2408	-0.1625	-0.1578	0.1460
Control 1	20	-0.874	-0.2107	-0.1941	-0.2451	-0.1830	-0.1563	0.1661
Control 2	49	-1.314	-0.2386	-0.1812	-0.2254	-0.1599	-0.1517	0.1702

the distributions of ndG in the three groups does not appear very different; both, location and dispersal measures are alike (Table AF1-10).

Even when the algorithm implemented in [13] will be detecting part of the necessary factors for the interaction of lncRNA with their targets, say the binding free energy for pairs of sequences, it is clear that this parameter even if necessary is not sufficient to predict real lncRNA / mRNA interactions.

Supplementary Figures

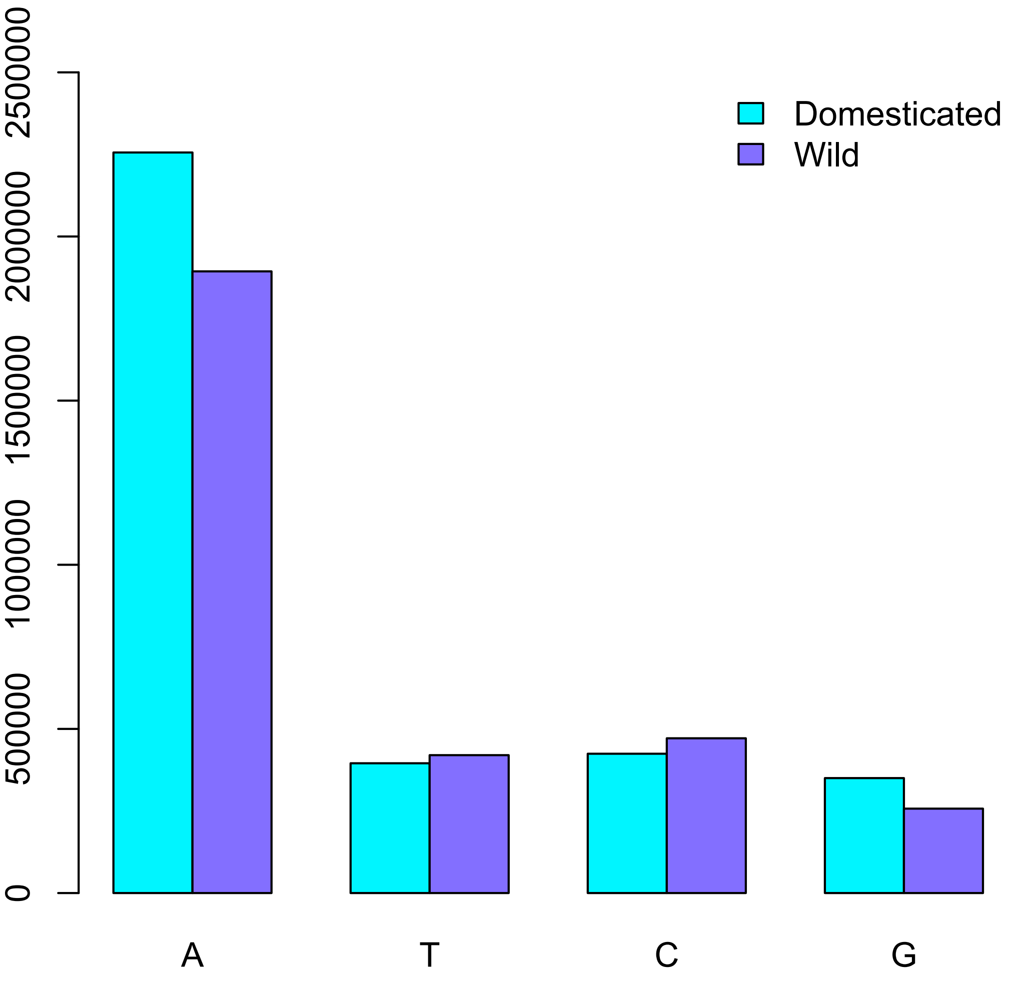

Figure AF1-2. **Number of reads of 24-nt length by each 5' terminal nucleotide.**

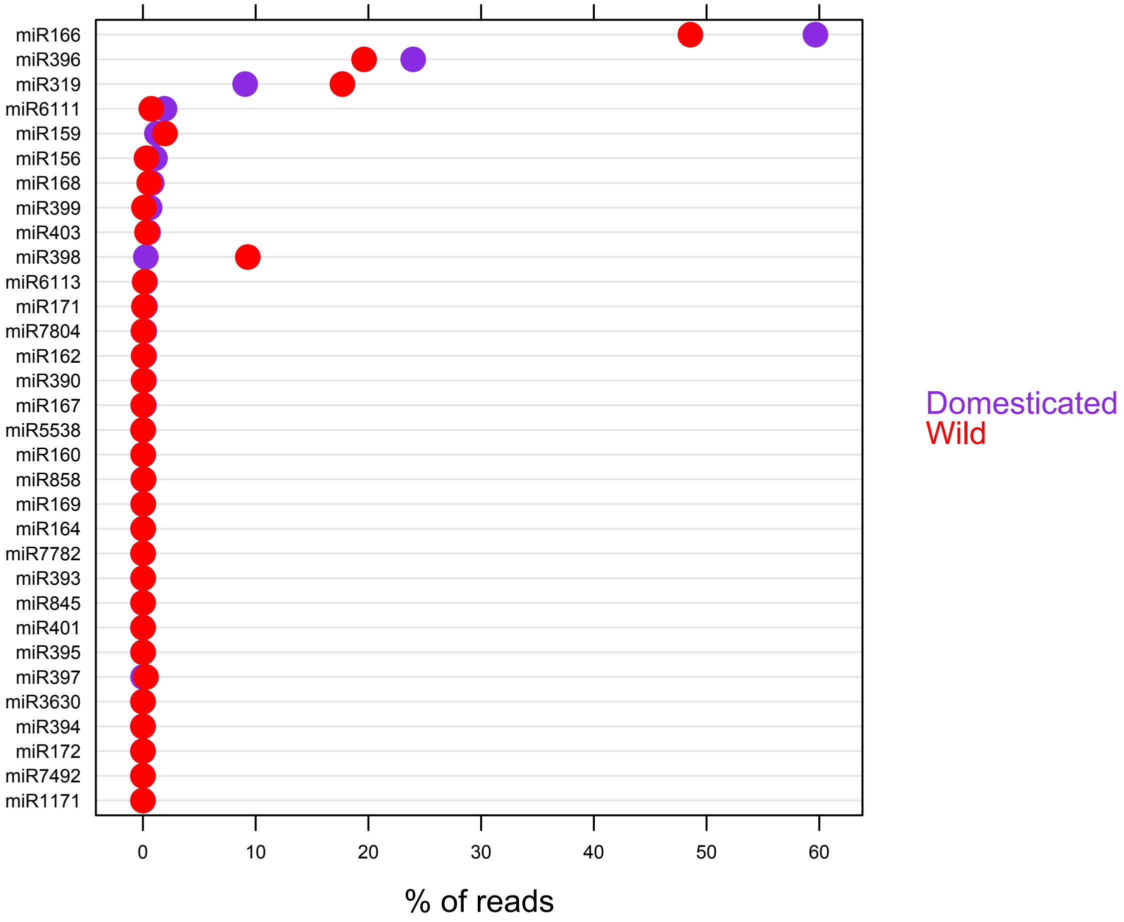

FIGURE AF1-3. **Families of miRNA identified in the meiocytes transcriptome.**

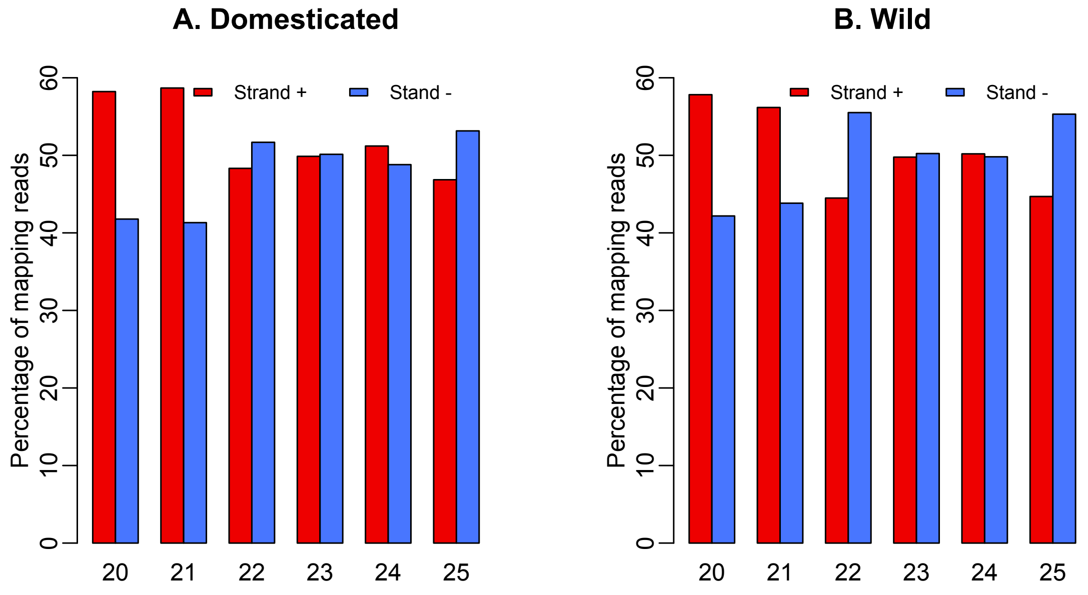

FIGURE AF1-4. **Mapping of sRNAs by strand.** (A) Reads mapped from domesticated genotype. (B). Reads mapped from wild genotype

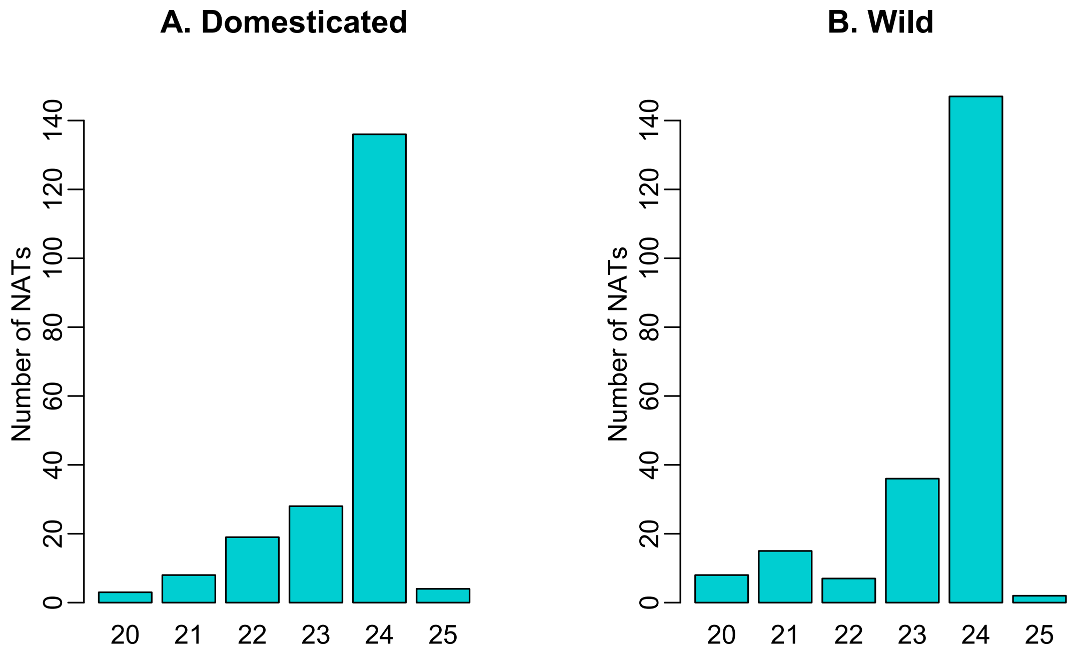

FIGURE AF1-5. **Number of natural antisense transcripts (NATs) that have sequence similarity with small RNAs by each sRNAs length.**

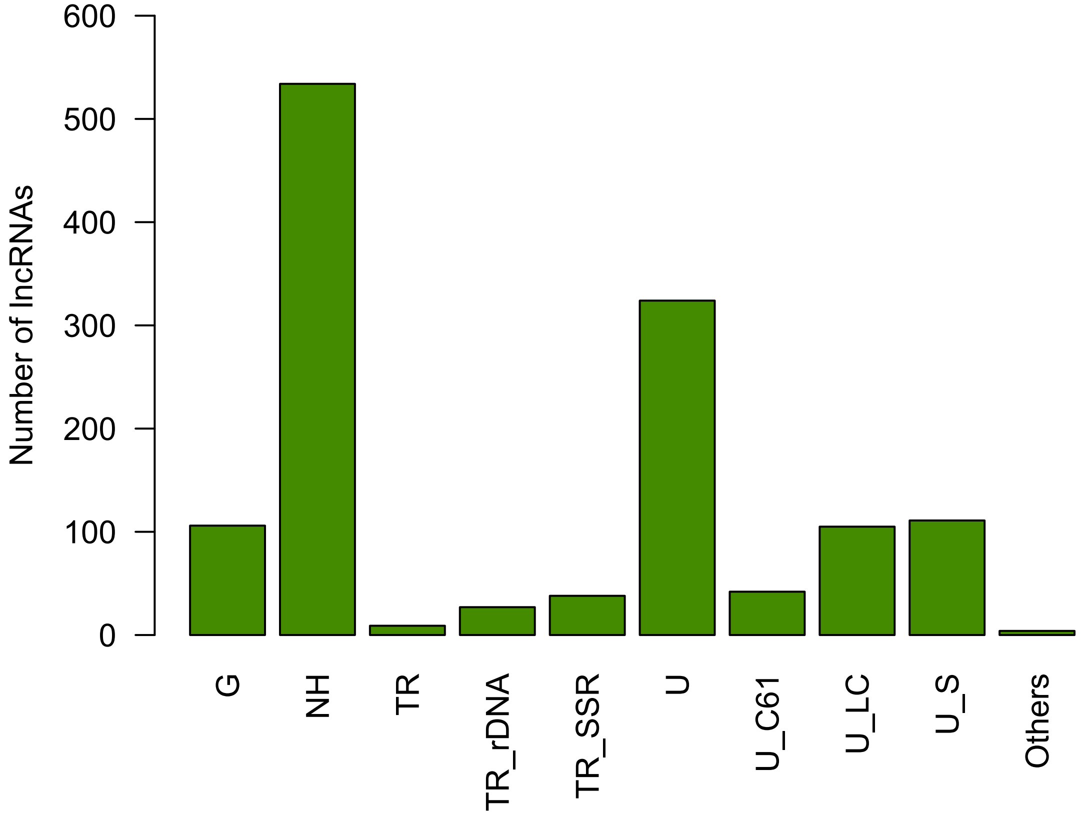

FIGURE AF1-6. **Repetitive elements (not TEs) found in the sunflower lncRNAs.** G = genes, NH=No hits found, TR = similar to known sunflower tandem repeats, TR_rDNA = ribosomal DNA, TR_SSR = microsatellites, U = similar to unknown sequences in the NCBI db, U_C61 = similar to contig 61, U_LC = unknown repeats (low complexity), U_S = similar to unknown repeats in the Sanger library [7].

Transposons (TEs) and Repetitive Elements (REs) in sunflower lncRNAs

To determine which of the lncRNAs reported here contained TEs or REs we performed a `blastn` analysis with three databases of repetitive elements, SunRep [20], RepBase [9] and Repeat Element Database (PGSB-REcat) [21]. Additionally, we employed the service in line of RepeatMasker.

For the `blastn` experiments our lncRNAs were compared with each one of the mentioned databases using parameters '`-dust no -evalue 1`' and results were carefully evaluated to determine the optimal threshold values to include only biologically relevant results, avoiding as much as possible false positives as well as false negatives. We center our analysis in the `bitscore` of the output that takes into account the length of the alignment, mismatches as well as gaps and that in contrast with the expected value (`evalue`) does not depend on the size of the explored database. We determined that a threshold of `bitscore` = 71 produced perfect alignments without mismatches or gaps of `length` = 38 bp. Thus we set a threshold of `bitscore` ≥ 70 in order to detect even small fragments of RE in the lncRNA. All the hits that passed the threshold `bitscore` ≥ 70 had an expected value `evalue` $< 1 \times 10^{-6}$ for all databases, thus the criterion for expected value is unnecessary, even if it is always fulfilled.

For the online service of RepeatMasker we uploaded our lncRNA sequences and compared with the full collection of *Arabodopsis thaliana*, using the best search engine (`cross_match`) with the maximum sensitivity ('slow' option).

Table AF1-11 presents the sources of data employed to detect TEs and repetitive elements in our sunflower lncRNAs and summarize the results obtained.

Table AF1-11. Number (n) and percentage (% of the 25,327 lncRNAs reported) by source of annotation.

Row	Source	Organism	Reference	Method	Threshold	n	%
1	SunRep	Sunflower	[20]	`blastn`	`bitscore` ≥ 70	3,470	13.70
2	RepeatMasker	*Arabodopsis*	Unpublished (*)	`cross_match`	Slow	209	0.83
3	RepBase	Eukaryotic	[9]	`blastn`	`bitscore` ≥ 70	17	0.07
4	PGSB-REcat	Plants	[21]	`blastn`	`bitscore` ≥ 70	14	0.06
5	Distinct lncRNAs containing TE or RE from one or more sources					3,626	14.32

* A.F.A. Smit, R. Hubley & P. Green, unpublished data. Current Version: open-4.0.5 (RMLib: 20140131 & Dfam: 1.3).

From Table AF1-11 we can see that, as expected, the largest number (and percentage) of lncRNAs detected to contain TE or RE elements is obtained with the database from sunflower elements ('SunRep'). However, the other sources for the analyses also gave small numbers of lncRNAs with TE or RE. In some cases the same lncRNA is significant for more than one source, thus row 5 of the table, presenting the total number and percentages of distinct lncRNAs with TE or REs is not equal to the sum of the values of n from rows 1 to 4. The following tables present a more detailed analysis of the results per source.

Table AF1-12 gives the number of lncRNAs with similarity to TE or REs elements at each one of the databases searched.

From Table AF1-12 we can see that a large number, 21,701 (85.683%), of lncRNAs do not present significant similarity with TE or REs elements in any of the searched databases (row 1), while the majority (3,550 of 3,626) of cases of lncRNAs with significant similarities are detected with only one DB (row 6 in Table AF1-12) and only in 2 lncRNAs the elements are detected in all four databases (row 13 in Table AF1-12). The largest number of lncRNAs detected in two datasets, 57 (row 9 in Table AF1-12) happens with databases SunRep and RepeatMasker (RepMar).

Table AF1-12. Cross-classification of the number of lncRNAs with similarity to TE or REs elements in the SunRep, RepBase, PGSB and RepeatMasker (RepMar) databases. 'n' gives the total number of lncRNAs per row, 'AvL' gives the average length of the lncRNAs in bp and 'DB' shows the number of databases where the lncRNAs in each row have similarities.

Row	n	%	AvL	SunRep	RepBase	PGSB	RepMar	DB
1	21,701	85.683	357	–	–	–	–	0
2	139	0.549	447	–	–	–	139	1
3	3	0.012	520	–	–	3	–	1
4	5	0.020	311	–	5	–	–	1
5	3,403	13.436	444	3403	–	–	–	1
6	3,550	14.017	Total of cases where DB=1					
7	7	0.028	311	–	7	–	7	2
8	1	0.004	290	–	1	1	–	2
9	57	0.225	602	57	–	–	57	2
10	4	0.016	673	4	–	4	–	2
11	1	0.004	225	1	1	–	–	2
12	70	0.277	Total of cases where DB=2					
11	1	0.004	308	–	1	1	1	3
12	3	0.012	551	3	–	3	3	3
13	2	0.008	397	2	2	2	2	4
14	3,626	14.318	Total of cases where DB> 0					
Total	25,327	100.000	370	3,470	17	14	209	–

Table AF1-13 presents the strength of evidence for classification of lncRNAs as containing TE or REs elements. This evidence is presented as the minimum (Min.) and average (Avg.) length of the alignments between lncRNAs and the corresponding TE or REs elements.

Table AF1-13. Minimum (Min.) and average (Avg.) lengths of significant alignment between lncRNAs and TE or REs elements in the SunRep, RepBase, PGSB and RepeatMasker (RepMar) databases obtained with `blastn` (SunRep, RepBase and PGSB) or RepeatMasker. Columns 'n', 'AvL' and '%' as in Table AF1-12.

				SunRep		RepBase		PGSB		RepMar	
Row	n	AvL	%	Min.	Avg.	Min.	Avg.	Min.	Avg.	Min.	Avg.
1	21,701	357	85.683	–	–	–	–	–	–	–	–
2	3	520	0.012	–	–	–	–	69	211	–	–
3	5	311	0.020	–	–	52	110	–	–	–	–
4	139	447	0.549	–	–	–	–	–	–	27	98
5	3,403	444	13.436	38	215	–	–	–	–	–	–
6	4	673	0.016	93	126	–	–	79	180	–	–
7	1	290	0.004	–	–	59	59	57	57	–	–
8	1	225	0.004	105	105	82	82	–	–	–	–
9	7	311	0.028	–	–	42	228	–	–	54	226
10	57	602	0.225	40	258	–	–	–	–	27	101
11	1	308	0.004	–	–	312	312	314	314	308	308
12	3	551	0.012	133	265	–	–	46	68	38	76
13	2	397	0.008	235	398	70	76	71	75	77	105

From Table AF1-13 we can observe that the absolute minimum for the length of alignment between lncRNA and TE or REs elements, 27 bp, happens for the procedure employed by RepeatMasker (rows 4

and 10, column 'RepMas' in Table AF1-13), while the minimum for the cases employing `blastn` (with threshold `bitscore` ≥ 70; columns 'SunRep', 'RepBase' and 'PGSB' in Table AF1-13) is equal to 38 bp in row 5 and column 'SunRep'. In general, the minimum and average lengths of the alignments show that our procedure is unlikely to produce false positives, because even small lengths of the lncRNAs containing TE or REs elements were detected.

qRT-PCR analysis of selected lncRNAs

Previously, our RNA-seq data for protein coding genes were validated for 5 genes by qRT-PCR, showing that fold changes in expression are consistent between RNA-seq and qRT-PCR in the domesticated meiocytes and somatic transcriptomes (see Figure 6 in [5]). Here we performed qRT-PCR analysis on selected lncRNAs to compare the estimated fold change in expression between the domesticated (D, HA89) and wild (W, Ac-8) genotypes. A total of 22 lncRNAs were selected for the analysis, and primers were designed using PRI3 online software, optimizing for size and conditions for qRT-PCR. Two lncRNAs were selected as controls, given their uniform expression in the two genotypes, as estimated by RNA-seq results.

qRT-PCR reactions were prepared and run as previously reported in [5] from RNA extracted from meiocytes from the domesticated (D, HA89) and wild (W, Ac-8) genotypes. All reactions were performed four times (technical replicates) with RNA from each source, and final results were analyzed by the $2^{-\Delta\Delta C_T}$ method [15]. Table AF1-14 summarize primary results for RNA-seq and qRT-PCR for the genes tested.

From Table AF1-14 we can see that only 18 of the 23 pairs of primers designed gave a unique specific PCR product to be analyzed by qRT-PCR (column SP), thus for the 5 rows in Table AF1-14 where there was not unique PCR product, all remaining qRT-PCR results are missing. Figure AF1-14 exemplifies cases where the PCR reaction gave a single specific product (panel A), or more than one product (Panel B). Given the fact that lncRNAs are, in general, expressed at absolute concentrations lower than protein coding genes [14], the number of PCR cycles needed to detect the product above the noise threshold (BE in Table AF1-14) is large and prone to errors [15]. In many cases this leads to undetermined values for the mean of the C_T cycle ('Und.' in the $\hat{E}\bar{C}_T$ columns in Table AF1-14); only genes with keys Con1, G1, G4, G5, G6, G8 and G10 (rows 7, 13, 16 to 18, 20 and 22 in Table AF1-14) have determined values in all columns, and thus can be used to compare RNA-seq with qRT-PCR results by the $2^{-\Delta\Delta C_T}$ method.

Table AF1-15 presents some of the possible comparisons of fold changes estimated by RNA-seq (F_R) and qRT-PCR (F_q) using the simple $2^{-\Delta C_T}$ comparison.

The first two rows of Table AF1-15 present the comparisons of fold changes for the control genes, Con1 and G1 between the D and W genotypes. From these data we can see that Con1 is a better control than G1, given that its fold change between the genotypes estimated by qRT-PCR is 1.7, while for G1 it is 0.25

For comparisons performed between genes in the wild genotype, it is important to consider that in RNA-seq the measurement in transcripts per million (TPM) is only valid when comparing the same gene in two or more conditions [24]. However, when comparing different genes in the same genotype (comparisons 'Within W' in Table AF1-15) TPM measurements can be biassed if the length of the genes are different, because longer genes have a greater probability of accumulating gene tags even if both genes compared are expressed at the same relative expression, thus only comparisons between genes of approximately the same size (column 'GS' in Table qt1) were performed in the comparisons 'Within W' in Table AF1-15. For those cases, all fold changes show the same tendency, even when large differences, as it is expected by the exponential nature of the qRT-PCR estimation [18].

Table AF1-16 presents the comparisons between $\log_2$ fold changes between the D and W genotypes performed by the $-\Delta\Delta C_T$ method with two independent controls (Con1 and G1).

TABLE AF1-14. **Results of RNA-seq and qRT-PCR for selected genes.** GS - Estimated gene size in bp; PS - Expected size of PCR product in bp; key - Internal key name; D and W relative expression (TPM) in domesticated (D) and wild (W) genotypes estimated from RNA-seq; SP - Single specific product?; BE - Baseline end cycle; $\hat{E}\bar{C}_T$ - Estimated efficiency times the estimated mean of C_T (threshold cycle; four technical replicates) for domesticated (D) and wild (W) genotypes; R^2 - Determination coefficient estimated for the standard curve calibration; Und. - Undetermined value.

				RNA-seq			qRT-PCR					
id	lncRNA	GS	PS	key	D	W	SP	BE D	BE W	$\hat{E}\bar{C}_T$ D	$\hat{E}\bar{C}_T$ W	R^2
1	c55101_g1_i1	547	180	C1	11	185	No	–	–	–	–	–
2	c57676_g1_i1	446	196	C2	17	329	No	–	–	–	–	–
3	c61042_g1_i1	741	194	C3	19	365	Yes	39	39	Und.	Und.	–
4	c63724_g1_i1	325	204	C4	20	496	Yes	39	39	Und.	Und.	–
5	c67445_g1_i1	839	202	C5	12	204	Yes	39	39	Und.	34.93	–
6	c31708_g1_i1	519	204	Con2	8	12	Yes	39	25	Und.	27.81	0.9989
7	c30256_g1_i1	509	153	Con1	4	4	Yes	26	26	29.89	29.05	0.9893
8	c37957_g1_i1	779	196	LF1_1	9	3	Yes	39	25	Und.	26.04	0.9936
9	c53383_g1_i1	533	204	LF1_2	8	2	No	–	–	–	–	–
10	c49261_g6_i2	1001	190	LD_2	58	10	No	–	–	–	–	–
11	c42595_g3_i1	1550	183	LD_1	19	3	Yes	39	35	Und.	36.80	0.9992
12	c49767_g4_i3	330	203	LD_3	17	1	Yes	39	29	Und.	31.82	0.9543
13	c34818_g1_i1	680	141	G1	1	1	Yes	27	29	43.5	45.5	0.9976
14	c24881_g1_i1	275	120	G2	0	30	Yes	39	39	Und.	Und.	–
15	c25421_g1_i3	963	147	G3	0	76	Yes	34	39	Und.	Und.	–
16	c35930_g1_i2	1048	149	G4	24	0	Yes	20	33	22.8	35.5	0.9998
17	c37781_g1_i1	807	157	G5	0	14	Yes	25	32	50.7	42.3	0.9562
18	c45086_g5_i2	1361	149	G6	0	96	Yes	30	31	59.3	56.5	0.9198
10	c46452_g1_i2	629	155	G7	1	143	Yes	39	39	Und.	Und.	–
20	c46901_g1_i3	2544	152	G8	15	0	Yes	19	26	20.2	26.7	0.9930
21	c53703_g1_i2	584	160	G9	31	0	No	–	–	–	–	–
22	c55492_g1_i1	440	154	G10	29	0	Yes	22	27	26.7	32.5	0.9999

TABLE AF1-15. Estimated fold changes by RNA-seq (F_R) and qRT-PCR using the $2^{-\Delta C_T}$ method (F_q) in distinct comparisons.

Between D and W		
Comparison	F_R	F_q
Con1 D *vs.* Con1 W	1.00	1.7
G1 in D *vs.* G1 W	1.00	0.25
**Within W**		
Comparison	F_R	F_q
Con1 *vs.* LD_3	4.0	6.8
Con2 *vs.* Con1	3.0	2.4
Con2 *vs.* LF1_1	4.0	3.4
C5 *vs.* LF1_1	68.00	474.00

In Table AF1-16 we can see that the tendency of the fold changes between the D and W genotypes for the five genes using qRT-PCR, $\log_2(F_q)$, and RNA-seq, $\log_2(F_R)$, are in general consistent when using two different control genes for the $-\Delta\Delta C_T$ method, even when, as expected the qRT-PCR measures

Table AF1-16. Comparisons of $\log_2$ fold changes for five genes by qRT-PCR using the $-\Delta\Delta C_T$ method with two independent controls (Con1 and G1), $\log_2(F_q)$ and by RNA-seq, $\log_2(F_R)$.

Control	Gene	$\log_2(F_q)$	$\log_2(F_R)$
Con1	G4	13.23	7.37
	G5	-7.87	-6.81
	G6	-2.25	-9.40
	G8	7.08	6.95
	G10	6.45	6.80
G1	G4	10.64	7.37
	G5	-10.46	-6.81
	G6	-4.84	-9.40
	G8	4.49	6.95
	G10	3.86	6.80

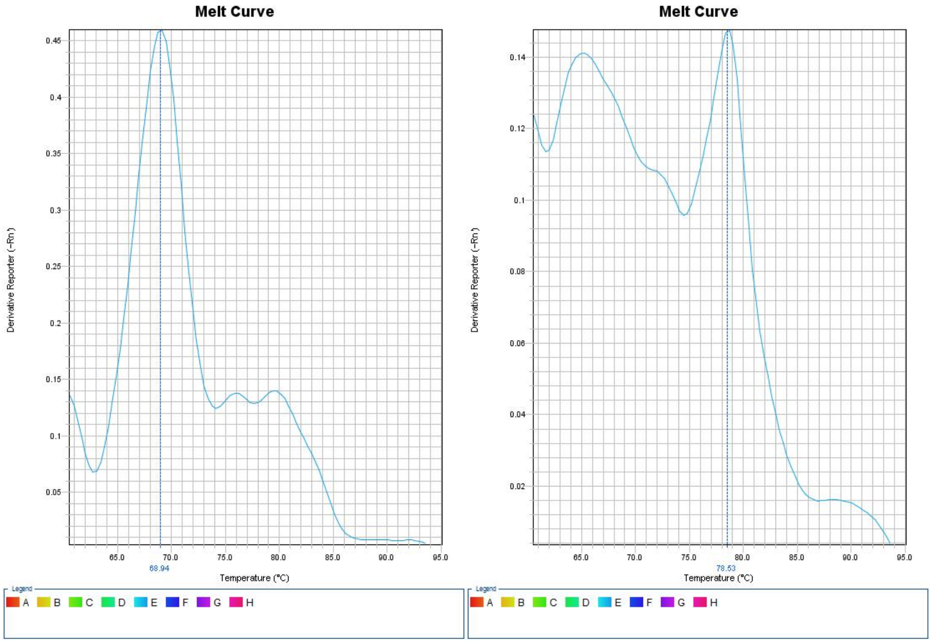

(A) Specific product for gene with key=‘Con1’. BE = 26.

(B) Not specific product for gene with key=‘C2’. BE = 33.

Figure AF1-7. Examples of melting curves for cases with specific (Panel A) and non-specific (Panel B) PCR products; see Table AF1-14 for keys of genes.

are more extreme and variable, given the exponential error structure [18] and low concentration of the lncRNAs.

References

[1] Coline Billerey, Mekki Boussaha, Diane Esquerré, Emmanuelle Rebours, Anis Djari, Cédric Meersseman, Christophe Klopp, Daniel Gautheret, and Dominique Rocha. Identification of large intergenic non-coding rnas in bovine muscle using next-generation transcriptomic sequencing. *BMC genomics*, 15(1):1, 2014.

[2] Sohini Chakraborty, Aritra Deb, Ranjan Kumar Maji, Sudipto Saha, and Zhumur Ghosh. Lncrbase: an enriched resource for lncrna information. *PloS one*, 9(9):e108010, 2014.

[3] Sven Diederichs. The four dimensions of noncoding rna conservation. *Trends in Genetics*, 30(4):121–123, 2014.

[4] Zhide Fang and Xiangqin Cui. Design and validation issues in rna-seq experiments. *Briefings in bioinformatics*, page bbr004, 2011.

[5] Nathalia M. V. Flórez-Zapata, M. H. Reyes-Valdés, Fernando Hernandez-Godínez, and Octavio Martínez. Transcriptomic landscape of prophase I sunflower male meiocytes. *Frontiers in Plant Science*, 5, June 2014.

[6] Luis Fernando García-Ortega and Octavio Martínez. How many genes are expressed in a transcriptome? Estimation and results for RNA-seq. *PloS one*, 10(6):e0130262, 2015.

[7] T Giordani, A Cavallini, and L Natali. The repetitive component of the sunflower genome. *Current Plant Biology*, 1:45–54, 2014.

[8] Murukarthick Jayakodi, Je W Jung, Doori Park, Young-Joon Ahn, Sang-Choon Lee, Sang-Yoon Shin, Chanseok Shin, Tae-Jin Yang, and Hyung W Kwon. Genome-wide characterization of long intergenic non-coding rnas (lincrnas) provides new insight into viral diseases in honey bees apis cerana and apis mellifera. *BMC genomics*, 16(1):680, 2015.

[9] Jerzy Jurka. Repbase update: a database and an electronic journal of repetitive elements. *Trends in genetics*, 16(9):418–420, 2000.

[10] Kriti Kaushik, Vincent Elvin Leonard, KV Shamsudheen, Mukesh Kumar Lalwani, Saakshi Jalali, Ashok Patowary, Adita Joshi, Vinod Scaria, and Sridhar Sivasubbu. Dynamic expression of long non-coding rnas (lncrnas) in adult zebrafish. *PloS one*, 8(12):e83616, 2013.

[11] Lei Kong, Yong Zhang, Zhi-Qiang Ye, Xiao-Qiao Liu, Shu-Qi Zhao, Liping Wei, and Ge Gao. Cpc: assess the protein-coding potential of transcripts using sequence features and support vector machine. *Nucleic acids research*, 35(suppl 2):W345–W349, 2007.

[12] Jianqin Li, Bin Wu, Jiang Xu, and Chang Liu. Genome-wide identification and characterization of long intergenic non-coding rnas in ganoderma lucidum. *PloS one*, 9(6):e99442, 2014.

[13] Jianwei Li, Wei Ma, Pan Zeng, Junyi Wang, Bin Geng, Jichun Yang, and Qinghua Cui. LncTar: a tool for predicting the RNA targets of long noncoding RNAs. *Briefings in bioinformatics*, 16(5):806–12, sep 2015.

[14] Xue Liu, Lili Hao, Dayong Li, Lihuang Zhu, and Songnian Hu. Long non-coding rnas and their biological roles in plants. *Genomics, proteomics & bioinformatics*, 13(3):137–147, 2015.

[15] Kenneth J Livak and Thomas D Schmittgen. Analysis of relative gene expression data using real-time quantitative pcr and the $2^{-\Delta\Delta C_T}$ method. *methods*, 25(4):402–408, 2001.

[16] Jie Lv, Wei Cui, Hongbo Liu, Hongjuan He, Youcheng Xiu, Jing Guo, Hui Liu, Qi Liu, Tiebo Zeng, Yan Chen, et al. Identification and characterization of long non-coding rnas related to mouse embryonic brain development from available transcriptomic data. *PLoS One*, 8(8):e71152, 2013.

[17] Jie Lv, Hongbo Liu, Zhijun Huang, Jianzhong Su, Hongjuan He, Youcheng Xiu, Yan Zhang, and Qiong Wu. Long non-coding rna identification over mouse brain development by integrative modeling of chromatin and genomic features. *Nucleic acids research*, page gkt818, 2013.

[18] John C Marioni, Christopher E Mason, Shrikant M Mane, Matthew Stephens, and Yoav Gilad. Rna-seq: an assessment of technical reproducibility and comparison with gene expression arrays. *Genome research*, 18(9):1509–1517, 2008.

[19] M Muthusamy, S Uma, S Backiyarani, and MS Saraswathi. Genome-wide screening for novel, drought stress-responsive long non-coding rnas in drought-stressed leaf transcriptome of drought-tolerant and-susceptible banana (musa spp) cultivars using illumina high-throughput sequencing. *Plant Biotechnology Reports*, 9(5):279–286, 2015.

[20] Lucia Natali, Rosa M Cossu, Elena Barghini, Tommaso Giordani, Matteo Buti, Flavia Mascagni, Michele Morgante, Navdeep Gill, Nolan C Kane, Loren Rieseberg, et al. The repetitive component of the sunflower genome as shown by different procedures for assembling next generation sequencing reads. *BMC genomics*, 14(1):686, 2013.

[21] Thomas Nussbaumer, Mihaela M Martis, Stephan K Roessner, Matthias Pfeifer, Kai C Bader, Sapna Sharma, Heidrun Gundlach, and Manuel Spannagl. Mips plantsdb: a database framework for comparative plant genome research. *Nucleic acids research*, 41(D1):D1144–D1151, 2013.

[22] Alejandro Padrón, Alvaro Molina-Cruz, Mariam Quinones, José MC Ribeiro, Urvashi Ramphul, Janneth Rodrigues, Kui Shen, Ashley Haile, José Luis Ramirez, and Carolina Barillas-Mury. In depth annotation of the anopheles gambiae mosquito midgut transcriptome. *BMC genomics*, 15(1):1, 2014.

[23] John L Rinn and Howard Y Chang. Genome regulation by long noncoding rnas. *Annual review of biochemistry*, 81, 2012.

[24] MD Robinson, DJ McCarthy, and GK Smyth. edger: a bioconductor package for differential expression analysis of digital gene expression data. *Bioinformatics*, 26(1):139–140, 2010.

[25] Marcel H Schulz, Daniel R Zerbino, Martin Vingron, and Ewan Birney. Oases: robust de novo rna-seq assembly across the dynamic range of expression levels. *Bioinformatics*, 28(8):1086–1092, 2012.

[26] Celia Schunter, Steven V Vollmer, Enrique Macpherson, and Marta Pascual. Transcriptome analyses and differential gene expression in a non-model fish species with alternative mating tactics. *BMC genomics*, 15(1):167, 2014.

[27] Jinyan Wang, Wengui Yu, Yuwen Yang, Xiao Li, Tianzi Chen, Tingli Liu, Na Ma, Xu Yang, Renyi Liu, and Baolong Zhang. Genome-wide analysis of tomato long non-coding rnas and identification as endogenous target mimic for microrna in response to tylcv infection. *Scientific reports*, 5, 2015.

[28] Liguo Wang, Hyun Jung Park, Surendra Dasari, Shengqin Wang, Jean-Pierre Kocher, and Wei Li. Cpat: Coding-potential assessment tool using an alignment-free logistic regression model. *Nucleic acids research*, 41(6):e74–e74, 2013.

[29] Tian-Zuo Wang, Min Liu, Min-Gui Zhao, Rujin Chen, and Wen-Hao Zhang. Identification and characterization of long non-coding rnas involved in osmotic and salt stress in medicago truncatula using genome-wide high-throughput sequencing. *BMC plant biology*, 15(1):131, 2015.

[30] Rosemarie Weikard, Frieder Hadlich, and Christa Kuehn. Identification of novel transcripts and noncoding rnas in bovine skin by deep next generation sequencing. *BMC genomics*, 14(1):1, 2013.

[31] Robert S Young, Ana C Marques, Charlotte Tibbit, Wilfried Haerty, Andrew R Bassett, Ji-Long Liu, and Chris P Ponting. Identification and properties of 1,119 candidate lincrna loci in the drosophila melanogaster genome. *Genome biology and evolution*, 4(4):427–442, 2012.

[32] Chunhui Yuan, Jingjing Wang, Andrew P Harrison, Xianwen Meng, Dijun Chen, and Ming Chen. Genome-wide view of natural antisense transcripts in arabidopsis thaliana. *DNA Research*, 22(3):233–243, 2015.

[33] Yu-Chan Zhang, Jian-You Liao, Ze-Yuan Li, Yang Yu, Jin-Ping Zhang, Quan-Feng Li, Liang-Hu Qu, Wen-Sheng Shu, and Yue-Qin Chen. Genome-wide screening and functional analysis identify a large number of long noncoding rnas involved in the sexual reproduction of rice. *Genome Biol*, 15(12):512, 2014.

[34] Changsong Zou, Qiaolian Wang, Cairui Lu, Wencui Yang, Youping Zhang, Hailiang Cheng, Xiaoxu Feng, Mtawa Andrew Prosper, and Guoli Song. Transcriptome analysis reveals long noncoding rnas involved in fiber development in cotton (gossypium arboreum). *Science China Life Sciences*, 59(2):164–171, 2016.
